# Efficacy of chemical sanitizers against *E. coli* O157:H7 in single- and multi-species biofilms under wet and dry conditions

**DOI:** 10.3389/fmicb.2026.1682881

**Published:** 2026-02-27

**Authors:** Kavitha Koti, Anna Macdonald, Argenis Rodas-Gonzalez, Tim McAllister, Claudia Narváez-Bravo

**Affiliations:** 1 Department of Food and Human Nutritional Science, University of Manitoba, Winnipeg, MB, Canada; 2 Department of Animal Science, University of Manitoba, Winnipeg, MB, Canada; 3 Agriculture and Agri-Food Canada, Lethbridge Research and Development Centre, Lethbridge, AB, Canada

**Keywords:** biofilms, sanitizers, spoilage bacteria, stainless steel, STEC, thermoplastic polyurethane

## Abstract

Effective sanitation of food-contact surfaces is essential for controlling *E. coli* O157:H7 in beef processing environments. This study evaluated how environmental conditions (temperature, humidity, and biofilm age), surface materials [stainless steel (SS) and thermoplastic polyurethane (TPU)], mechanical action, and sanitizer type influence the survival of *E. coli* O157:H7 and spoilage bacteria within single- and mixed-species biofilms. Biofilms were formed on TPU and SS coupons at 10 °C or 25 °C, stored under wet 60–90% relative humidity (RH) and dry (20–50% RH) conditions at 10 °C and 25 °C for up to 60 days, and then exposed to detergents, a group was scrubbed other was not, then exposed to sanitizers quaternary ammonium compounds (Quats), sodium hypochlorite (Shypo), sodium hydroxide (SHyd), hydrogen peroxide (Hyp), peroxyacetic acid (PeroA) or BioDestroy^®^. Results showed that sanitizer efficacy was strongly influenced by the interaction of biofilm age, scrubbing, and sanitizer type (*p <* 0.01). Across treatments, biocides achieved greater reductions on SS (up to 7 log) than on TPU (up to 5.6 log). BioDestroy^®^, specifically formulated to eradicate biofilms, was the most effective sanitizer. ATP bioluminescence testing revealed that scrubbing markedly reduced organic residues, lowering RLU values from >14,000 on non-scrubbed surfaces to ~100–180 on scrubbed surfaces. Mixed-species biofilms containing *Carnobacterium* and *Lactobacillus,* in combination with scrubbing, showed the greatest reduction of *E. coli* O157:H7. However, conditions such as mature or dry biofilms, lack of mechanical action, and treatment with hydrogen peroxide allowed *E. coli* O157:H7 survival, reflecting the structural resilience of biofilms and the pathogen’s genetic tolerance to oxidative stress. Notably, *E. coli* O157:H7 was not detected on TPU or SS after 60 days of storage under either wet or dry conditions. Spoilage bacteria varied in resilience: *Comamonas* and *Raoultella* were harder to control on TPU, and *Pseudomonas aeruginosa* was most resistant, especially in wet TPU biofilms at 10 °C.

## 1 Introduction

Approximately 474 cases of foodborne illnesses linked to O157:H7 are reported annually in Canada (PHAC, 2020). Of the Shiga toxigenic *Escherichia coli* (STEC) serogroups, O157:H7 is still the most frequent serotype associated with foodborne outbreaks and food recalls (Terajima et al., 2017). *E. coli* O157:H7 is notorious for causing severe illness, including bloody diarrhea and life-threatening complications such as hemolytic-uremic syndrome (Rahal et al., 2012; Jangid et al., 2024). STEC has been linked to recalls and outbreaks from various food sources, including beef, produce, raw dairy products and flour (Baylis, 2009; Narvaez-Bravo et al., 2013; Gill et al., 2020; Marshall et al., 2020). Foodborne pathogens can survive in food processing facilities and may persist in the environment if they are not effectively removed by routine cleaning and sanitation procedures. Biofilm formation is a key bacterial survival strategy, as it protects against environmental stresses such as antibiotics, sanitizers, and desiccation (Donlan, 2002). Previous research has reported that temperature and humidity influence biofilm formation on various surfaces, including rubber, glass, stainless steel, and plastic. Polyurethane (PU) encompasses a broad class of polymers that can be either thermoset or thermoplastic. Among these, thermoplastic polyurethane (TPU) is valued in the food industry for its flexibility, durability, resistance to oils and fats, and ease of cleaning (Restivo et al., 2024). Conveyor belts manufactured from TPU and stainless steel (SS) surfaces are among the most common food-contact materials in processing facilities. Stainless steel has been extensively studied due to its widespread use in food equipment and its well-documented susceptibility to biofilm colonization (Visvalingam et al., 2017; Wang, 2019). In contrast, conveyor belts made from PU incorporate various material combinations, which may introduce variability in microbial colonization. Previous studies have shown that TPU can support greater bacterial biofilm formation than SS (Veluz et al., 2012). Therefore, evaluating TPU, widely used in beef processing, alongside stainless steel, is essential for accurate risk assessment and will provide valuable insight into how different types of TPU influence microbial colonization and cleaning effectiveness.

Cleaning and sanitation procedures are critical for reducing microbial loads and ensuring food safety (CFIA, 2020). These procedures typically begin with dry cleaning to remove visible organic matter, followed by rinsing with hot water (~45 °C) to eliminate residual debris. Detergents are then applied, and surfaces are scrubbed to ensure thorough cleaning. Because the presence of organic material can significantly reduce the efficacy of sanitizers, effective removal of organic matter is essential for proper sanitation (Chen et al., 2024). Failure to completely remove organic residues, particularly in hard-to-reach areas such as behind conveyor belts or when cleaning is limited by time constraints, allows microorganisms to persist, attach, and form biofilms on food-contact surfaces. Over time, older dry biofilms can rehydrate and resume development, serving as reservoirs for cross-contamination (Adator et al., 2018; Wang et al., 2018). Compounding the issue, biocide concentrations recommended by manufacturers are typically based on the susceptibility of planktonic bacteria rather than sessile cells within mature biofilms (Koti et al., 2024). Therefore, it is essential to evaluate whether biocide concentrations are effective against established biofilms. This research assessed the efficacy of six biocidal solutions, sodium hypochlorite, sodium hydroxide, two types of quaternary ammonium compounds (first- and third-generation Quats), hydrogen peroxide, and peroxyacetic acid, in reducing *E. coli* O157:H7 biofilms under varied conditions. Wet and dry biofilms were formed on SS and TPU surfaces at 10 °C and 25 °C and stored for 6, 30, and 60 days. The impact of mechanical scrubbing on biofilm removal and bacterial survival was also evaluated. The study objectives were to (i) determine how environmental (temperature, incubation time) and management (surface material, surface dryness, biofilm removal, and sanitizer type) factors influence the reduction of *E. coli* O157:H7 within biofilms on food-contact surfaces; (ii) compare the effectiveness of different sanitizers and evaluate how multispecies spoilage biofilms affect *E. coli* O157:H7 reduction under similar conditions; and (iii) assess the relative cleanliness of food-contact surfaces through ATP testing following biocide exposure, and evaluate *E. coli* O157 survival after biocide treatment.

## 2 Methods

### 2.1 Bacteria strain selection and culture conditions

A total of six bacterial strains were used in this study: O157:H7 strain (R508), a strong biofilm producer positive for curli and cellulose, two lactic acid bacteria (LAB), and two spoilage bacteria (SP) combinations, *Comamonas koreensis + Raoultella terrigena* and *Pseudomonas aeruginosa + C. koreensis* (Table 1). All bacterial strains were obtained from the Culture Collection of the Department of Food and Human Nutritional Sciences at the University of Manitoba. Strain selection was based on their biofilm formation abilities, the nature of their interactions (neutral, synergic and antagonistic) when combined with O157:H7 and the bactericidal and biofilm eradication concentrations identified through 96-well microplate assays (Koti et al., 2024). The bacterial cultures were maintained in Trypticase Soy Broth (TSB; Becton, Dickinson and Company, MD, United States) supplemented with 15% glycerol and stored at −80 °C. Stock cultures were plated on trypticase soy agar plates (TSA; Becton, Dickinson and Company, MD, United States), and O157:H7 strains were cultured on MacConkey agar plates (Hardy Diagnostics Inc., Santa Maria, CA, United States). A single colony was transferred from each plate into a 5 mL tube of TSB and incubated at 37 °C for 18 to 24 h. Cells were harvested by centrifugation (4,500 x G for 5 min. at room temperature). After centrifugation, the supernatant was decanted, and the pellet was resuspended in 5 mL sterile Butterfield’s Phosphate Buffer (BPB) (Hardy Diagnostics Inc., Santa Maria, CA, United States). This procedure was repeated three times. Finally, the pellet was suspended in a tube at 10^9^ CFU/mL in 10 mL of sterile BPB. Each bacterial suspension was adjusted to a final concentration of 10^8^ CFU/mL using a 0.5 McFarland standard and further diluted in LB-NS broth (LB-NS; Tryptone 10 g/L and yeast extract 5 g/L) supplemented with 16.7% filter sterilized beef purge (10% v/v) as previously described (Nan et al., 2022) to obtain a final population of 10^6^ CFU/mL (Poimenidou et al., 2016).

**Table 1:** STEC, LAB and spoilage bacteria used in this study.

Serotype	Strain ID	Source	Category
O157:H7	R508	Bovine/faeces	STEC
*Lactobacillus bulgaricus*	ATCC11842	Yogurt	LAB
*Carnobacterium piscicola*	M5L1	Vacuum package pork	LAB
*Comamonas* sp.	25_64	Meatpacking plant	Spoilage
*Raoultella* sp.	ENT25_16	Meatpacking plant	Spoilage
*Pseudomonas aeruginosa*	ATCC 7700	Well water	Spoilage

### 2.2 Culture combination

Three strain-combinations were used to form multispecies biofilms on food contact surfaces, which included: one LAB combination, T1 (neutral): *Carnobacterium piscicola + Lactobacillus bulgaricus* + O157:H7 R508 and two spoilage (SP) bacteria combinations, T2 (synergistic): *Comamonas koreensis + Raoultella terrigena* + O157:H7 R508 and T3 (antagonistic): *Pseudomonas aeruginosa + C. koreensis* + O157:H7 R508. *E. coli* O157:H7 (R508), a strong biofilm former with positive curli and cellulose phenotypes, was included for its robust biofilm-forming capacity. Two lactic acid bacteria (LAB), *Carnobacterium piscicola* and *Lactobacillus delbrueckii* subsp. *bulgaricus* ATCC 11842, were selected due to their ability to form biofilms without exhibiting antagonistic effects against *E. coli* O157. Additionally, combination T2 was included as previous findings demonstrated that this biofilm combination exhibits synergistic effects with *E. coli* O157. *Pseudomonas aeruginosa* was also selected due to its prevalence as a spoilage bacterium in food processing environments, its robust biofilm-forming capacity (Thi et al., 2020) and its known tolerance to disinfectants (Sagripanti and Bonifacino, 2000).

### 2.3 TPU and SS coupons preparation

The same surface types and preparation steps were used for single-species *E. coli* O157:H7 and multispecies biofilms to ensure comparability across experiments.

TPU coupons were prepared by cutting a 2-ply white urethane smooth-top food-grade conveyor belt (FDA-approved for food contact; 2E8U 0/02 White, NuTech Conveyor Components, Milton, Canada) into 2 × 2 cm pieces. Before use, coupons were sanitized overnight in 70% hydrogen peroxide (Accel^®^ PREVention^™^/MC, Diversey^™^) as described by Nan et al. (2022).

SS (type 304, 2B finish, both sides 2 cm-diameter; Pegen Industries Inc., Stittsville, Canada) were washed with distilled water and sonicated (Branson 2,800, Branson Ultrasonics Co., Danbury, CT, United States) in aqueous phosphoric acid (15% v/v) for 20 min at 60 °C. After sonication, coupons were rinsed with water and allowed to dry. Coupons were then dry sterilized in an autoclave before use (Adator et al., 2018; Nan et al., 2022).

### 2.4 Multispecies biofilm formation

Multispecies biofilms were prepared as described previously (Nan et al., 2022). Briefly, sterile coupons were transferred to sterile Petri dishes (60 ×15 mm; VWR^™^, Radnor, PA, United States) and SP-mixed bacterial cultures (T1: *Carnobacterium piscicola + Lactobacillus bulgaricus*, T2: *Comamonas koreensis + Raoultella terrigena*; T3: *Pseudomonas aeruginosa + C. koreensis*) were diluted in LB-NS broth supplemented with 16.7% filter sterilized beef purge (10% v/v) to achieve a final population of 10^6^ CFU/mL. Cultures were mixed, and 5 mL of the mix was added to each coupon. Coupons were placed at either 10 °C or 25 °C for 6 d to form biofilms. Biofilms were incubated in a temperature-controlled room maintained at 23–25 °C. For the 10 °C condition, coupons were kept in a refrigerated unit with controlled temperature. T1, T2 and T3 combinations were allowed to establish biofilms for 6 days because most of the selected require at least 6 days of incubation to develop mature biofilms. On d 6, coupons were washed three times (10 mL/coupon) with Butterfield’s Phosphate Buffer (BPB) and transferred to a new sterile Petri dish. Then, a fresh O157:H7 culture was diluted in LB-NS broth with beef purge to achieve 10^3^ CFU/mL and introduced onto preformed biofilms on coupons. The coupons were incubated at 10 °C and 25 °C for an additional 6 d. After incubation, the coupons were washed with BPB three times (10 mL BPB/coupon) and then placed in new sterile Petri dishes. Controls consisted of single-species O157:H7 and SP combinations (T1, T2, and T3) without O157:H7. This approach was designed to simulate food-processing environments, where spoilage organisms are often already established on surfaces. Introducing *E. coli* O157:H7 after the spoilage biofilm matured reflects a realistic contamination scenario, in which pathogenic *E. coli* becomes incorporated into pre-existing multispecies biofilms rather than initiating biofilm formation alone. Negative controls were not inoculated and contained only mLB-NS. Coupons were stored in sealed plastic containers along with a digital thermometer plus a humidity meter. For wet biofilms, coupons were stored in moist conditions [60–90% relative humidity, (RH)]. Dry biofilms were stored at relative humidity (RH) levels ranging from 20 to 50%. 165-166Groups of wet and dry biofilms were assigned to the two respective temperatures and stored for 6, 30 and 60 d. These time points were selected to represent fully established (6 d) and long-term persistent biofilms (30 and 60 d) that food-processing environments could encounter. For wet biofilms, to maintain moisture, they were sprayed with sterile water once daily (150 μL/coupon). The coupons were subjected to cleaning and disinfection at 6, 30 and 60 d using procedures that closely reflected practices employed in processing facilities. The procedure involved rinsing the coupons with hot water (60 °C) and applying detergent (50 °C), followed by mechanical scrubbing with sterilized brushes (Oxo Deep Clean Brush Set, USA). A set of coupons was designated as “scrubbed coupons,” where organic material was removed mechanically using brushes. Scrubbing was performed manually on both sides of the coupons using a zigzag and up-and-down motion, applying moderate pressure for 30 s. A non-scrubbed group received only detergent on the surfaces for 60 s. Following detergent application, all coupons were rinsed with 60 °C sterile distilled hot water (10 mL/coupon) and placed in a new sterile Petri dish. A set of coupons (scrubbed and non-scrubbed) was tested for ATP as an indicator of cleanliness using Clean-Trace surface swabs (3 M Co., St. Paul, MN).

### 2.5 Biocide and detergent solutions

Six disinfectants commonly used in the food industry were evaluated according to the manufacturer’s recommendations (see Supplementary Table S1). HC-10 detergent (ECOLAB HC-10 General purpose chlorinated manual cleaner) was prepared by adding 15 g of HC-10 per 1 L of warm potable water. All the stock solutions were prepared in hot water at 50 °C and maintained at the same temperature in a water bath. The solutions were used within 30 min of preparation. Before initiating testing, the concentration of total available chlorine in the sodium hypochlorite solution was tested using a chlorine analysis Test Kit (HACH, Model CN-65, Ontario, Canada).

### 2.6 Assessment of biocidal activity on single- and multi-species biofilms and *E. coli* O157 survival

The eradication concentrations for single- and multi-species *E. coli* O157:H7 biofilms were determined in our previous work on 96-microplate wells format (Koti et al., 2024). Based on those findings, and in alignment with industry practices, the concentrations recommended by the manufacturers were selected for use in this section (Supplementary Table S1). For certain chemicals, such as sodium hypochlorite and quaternary ammonium compounds (Quats), the higher recommended concentration was used, as the lower concentration proved insufficient for effective biofilm removal. The biocides tested included sodium hypochlorite (Shypo, 1,200 ppm), sodium hydroxide (Shyd, 2,500 ppm), Quat’s Powerquat (PQ, 550 ppm), Quats Germarc (GM, 600 ppm), hydrogen peroxide (250 ppm), and peroxyacetic acid alcohols (Biodestroy, PAA, 600 ppm) (Table 2). Concentrations were prepared according to manufacturer’s recommendations (Supplementary Table S1). Coupons containing biofilms were immersed in 10 mL of each biocide solution and exposed for 2 min. Following treatment, the coupons were rinsed with 10 mL of hot water (60 °C) to remove residual disinfectant. Each coupon was then placed into a sterile homogenizing bag containing 5 mL of Dey/Engley broth and allowed to stand for 5 min.

**Table 2:** Correlation between environment and management conditions traits with count reduction.

Environmental and management factors	Correlation with microbial count reduction (r)
Biofilm mechanical remotion	−0.146**
Duration of storage	−0.537**
Temperature	−0.323**
Biofilm surface dryness	−0.010 ns
Coupon material	0.264**
Sanitizer	−0.006 ns

Biofilm mechanical removal: scrubbed vs. no scrubbed. Storing day: 6, 30, 60 days. Temperature: 10 °C vs. 25 °C. Biofilm surface dryness: dry vs. wet. Coupon material: stainless steel (SS) surfaces vs. polyurethane (TPU). Sanitizer: S1 = PAA (Organic peroxy acids or BioDestroy^®^) 600 ppm; S2 = Quats (GM) 600 ppm; S3 = Sodium hydroxide (SHyd) 2,500 ppm; S4 = Hydrogen peroxide (HyP) 250 ppm; S5 = Quats (PQ) = 550 ppm; S6 = Sodium hypochlorite (Shypo) 1,200 ppm. ns = no significant, *p* > 0.05; *p < 0.05; **p < 0.01. Principal component analysis.

For bacterial enumeration, SS and TPU coupons were sonicated at 40 kHz for 1 min (Branson 2,800, Branson Ultrasonics Co., Danbury, CT, United States). Ten-fold serial dilutions were prepared in microcentrifuge tubes. Five drops (10 μL per drop) were dispensed in duplicate onto MacConkey and MRS agar (Difco^™^ Lactobacilli MRS Agar, BD Diagnostics, USA) overlaid with 10 mL of TSA. MacConkeyTSA and MRS agar were used to culture STEC, spoilage (SP) and lactic acid bacteria (LAB), respectively.

To assess *E. coli* O157 survival, samples that did not produce detectable colonies (<10 CFU/mL) after plating were subjected to enrichment in modified trypticase broth (mTSB) (Oxoid Dehydrated Culture Media, mTSB, USA) for 24 h at 37 °C. After incubation, the mTSB was plated on MacConkey and incubated for 24 to 48 h at 37 °C (Reed and Reed, 1948; Herigstad et al., 2001; Chen et al., 2003; Naghili et al., 2013). All experiments were conducted in three independent biological replicates, each with three technical replicates.

### 2.7 ATP testing

ATP testing was included to determine whether it would assist in detecting organic matter from biofilms. Random samples were collected, aiming to swab coupons with higher contamination levels, where ATP testing could provide more informative results regarding sanitation efficacy and the presence of organic matter. Negative controls consisted of coupons only treated with LB-NS broth. Sets of TPU and SS coupons stored at different times were swabbed in north-to-south and east-to-west directions while rotating the swabs and applying slight pressure. The test was activated immediately after sample collection by mixing the sample with the reagents in the collection device and then shaking it side to side for approximately 5 s. Next, ATP levels were measured with the 3 M Clean-Trace NG Luminometer and recorded in relative light units (RLU). A group of coupons was prepared to evaluate microbial reduction after exposure to biocides, immediately following the cleaning procedure. Coupons containing biofilms were immersed in 10 mL of each biocide for 2 min and then rinsed with 10 mL of hot water (60 °C). After rinsing, coupons were placed into sterile bags (207 mL, Nasco^™^ Whirl-Pak^™^) containing 5 mL of Dey/Engley neutralizer broth and allowed to stand for 5 min. The coupons were then sonicated at 40 kHz for 1 min (using a Branson 2,800, Branson Ultrasonics Co., Danbury, CT, United States). Finally, bacterial colonies were enumerated using the drop plate method (Herigstad et al., 2001; Naghili et al., 2013).

The ATP levels were categorized based on RLU thresholds: 0–10 RLU as excellent, 10–30 RLU as good, and 30–100 RLU as marginal. In the meat processing industry, RLU values below 100 are typically required for food-contact surfaces, particularly in raw ground beef processing areas. The pass/fail limit in this study was based on the instrument manufacturer’s guidelines and previous research, and anything above 100 RLU is considered to be a fail in the meat industry (Kupski, 2010; Osimani et al., 2014; Sogin et al., 2021). In the meat processing industry, particularly in raw ground beef processing areas, RLU values below 100 are generally considered the upper threshold for acceptable cleanliness, although values closer to 10 RLU are preferred to ensure minimal organic residue.

### 2.8 Statistical analysis

Data were analyzed using SAS, Version 9.4, by SAS Institute Inc., Cary, NC (SAS, 2012). To determine the environmental and management conditions affecting biofilm development of *E. coli* O157:H7 alone (Objective one), the nominal categorical variables (i.e., biofilm mechanical removal, coupon material, biofilm surface dryness, sanitizer) were converted into a numerical format by using one-hot categorical data encoding (e.g., 0100). This technique is applied when the categories have no inherent order or ranking. Correlation analyses were then performed to identify the influence of the main variables on pathogen numbers. For correlation analysis (PROC CORR), the Pearson correlation coefficient was used for the variables temperature and day, while the Spearman rank coefficient (rs) was used for mechanical removal of biofilms, coupon material, biofilm surface dryness, and sanitizer.

Principal component (PC) analysis was performed on the environment (temperature, incubation time) and management (surface material, surface dryness, biofilm removal, and sanitizer type) variables that significantly correlated with count reduction using SAS PROC FACTOR (SAS, 2012). Three PC were retained, and their correlation coefficients were plotted to evaluate relationships in each sanitizer. Based on the PC analysis results from O157:H7 single-species biofilms, sanitizers were evaluated under similar environmental and management conditions that showed the most significant reductions, specifically at 6 d of incubation, at 10 °C and 25 °C, on both TPU and SS surfaces, with and without mechanical scrubbing, under both wet and dry conditions. For objective two, eight combined conditions were selected based on these findings to assess the impact of spoilage and lactic acid bacteria (LAB) on O157:H7 within multispecies biofilm combinations (T1, T2, and T3). Data were analyzed using the PROC MIXED procedure within SAS (Cary, NC) version 9.4 (SAS, 2012) under a completely randomized factorial design. Sanitizer, biofilm surface dryness, environmental, and management conditions, as well as their interactions, were considered as fixed effects, while repetition was considered a random effect. Pathogen and spoilage bacterial populations at inoculation were included as covariates in the model. Differences among means were determined using the least significant difference (LSD) test at the 5% significance level.

For the third objective, frequency analysis (PROC FREQ) was used to determine the number and percentage of O157:H7 survival in single-species and multispecies biofilms after biocide application at different environmental and management conditions. Additionally, the central tendency for ATP testing was measured by the arithmetic mean (PROC MEAN).

In summary, the statistical analyses included correlation testing (Pearson and Spearman) and principal component analysis to identify key environmental and management drivers of biofilm reduction using data from O157:H7 single-species biofilms. Mixed-model analyses were applied for multispecies biofilms under a factorial design, and frequency and descriptive analyses were used to assess survival patterns and ATP trends. Together, these approaches allowed us to evaluate the individual and combined effects of environmental conditions, surface properties, mechanical removal, and sanitizer type on O157:H7 biofilm behaviour across all objectives.

## 3 Results and discussion

### 3.1 Environmental and procedural factors impacting *E. coli* O157:H7 reduction

#### 3.1.1 Correlation among environmental and management factors and count reduction

Duration of storage showed the strongest negative correlation with count reduction (r = −0.537, *p <* 0.01), indicating that longer storage time was associated with a lesser reduction in O157 (Table 2). This finding aligns with previous research indicating that mature biofilms exhibit increased EPS production and structural complexity, enhancing their resistance to antimicrobial agents (Simões et al., 2006). Similarly, temperature was negatively correlated with O157 reduction (r = −0.323, *p <* 0.01), with biofilms formed at 25 °C displaying greater resilience than those at 10 °C. Elevated temperatures likely promote EPS production and biofilm density, which impede sanitizer penetration (Ryu and Beuchat, 2005). Scrubbing compared to no scrubbing showed a negative correlation with O157 survival (r = −0.146, *p <* 0.01), suggesting that scrubbing improved O157 reduction. The coupon surface was positively correlated (r = 0.264, *p <* 0.01), with reductions in O157 reduction being greater for SS than TPU. This likely reflects the smoother, less hydrophobic nature of SS, which inhibits biofilm adhesion and enhances sanitizer efficacy (Donlan, 2000; Sinde and Carballo, 2000). No significant correlations were found between biofilm humidity conditions (dry vs. wet) and the type of sanitizer (Table 2), suggesting these factors alone are less critical compared to biofilm age, temperature, mechanical removal, and surface material.

#### 3.1.2 Principal component analysis (PCA) for each sanitizer

While correlation analysis identified the strength and direction of individual relationships between variables, it could not capture the combined and interactive effects of multiple factors influencing *E. coli* O157 reductions. The PCA revealed that the first three PCs collectively explained 74% of the standardized variance, with PC1, PC2, and PC3 accounting for 34, 20, and 20%, respectively (Table 3). Variables strongly associated with PC1 included mechanical removal, biofilm storage duration, temperature, coupon material, and count reduction (based on the largest loading values). PC2 was mainly influenced by mechanical removal, while PC3 highlighted the role of coupon material, along with moderate contributions from storage duration and temperature.

**Table 3:** Principal component loadings (74%).

Variables	PC1	PC2	PC3
Eigen value	1.683	1.0	1.0
Proportion	0.34	0.2	0.2
Biofilm mechanical remotion	0.238	0.966	0.0
Storing day	0.669	0.196	0.309
Temperature	−0.474	0.139	0.218
Coupon material	0.335	−0.098	0.926
Count reduction	0.917	0.0	0.0

**Figure 1:**
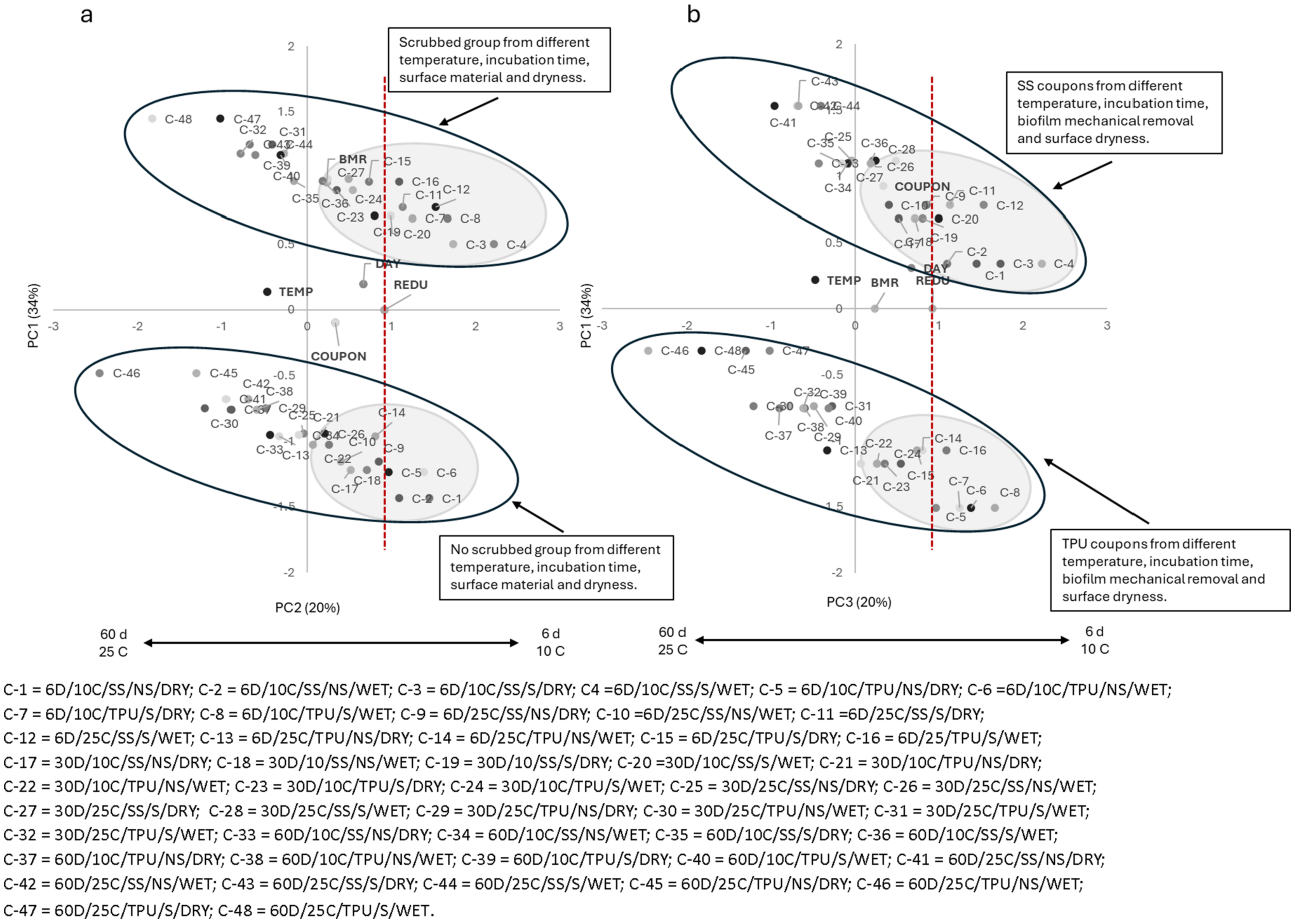
**(a)** Plotting principal component (PC1 × PC2) analysis for biofilm pathogen reduction (REDU) under different environment and management conditions when *organic peroxy acids sanitizer (PAA)* was applied. **(b)** Plotting principal component (PC1 × PC3) analysis for biofilm pathogen reduction under different environments and management conditions when *organic peroxy acids sanitizer (PAA)* was applied. Grey-shaded regions indicate sample groupings, and the red vertical line represents the landmark for count reduction. Factors positioned on the positive side of PC1 and near or to the right of the landmark suggest *E. coli* 0157: H7 susceptibility to PAA. Storing day (DAY): 6 = 6D, 30 = 30D, 60 = 60D days. Temperature (TEMP): 10 °C vs. 25 °C. Coupon material (COUPON): stainless steel = SS, polyurethane = TPU. Biofilm mechanical removal (BMR): scrubbed = S, no scrubbed = NS.

For PC1 × PC2 For the organic peroxy acid sanitizer (S1 = PAA), a clear segregation between scrubbed samples (top; positive side of PC2) and non-scrubbed samples (bottom; negative side of PC2) was observed (Figure 1A). In both groups, most samples stored for 6 days at 10 °C, regardless of biofilm surface dryness or coupon material, were located on the positive side of PC1 and clustered near the landmark of count reduction, irrespective of biofilm surface dryness or coupon material. These findings indicate that, when O157:H7 is present on surfaces at 10 °C, it was susceptible to sanitizers; but mechanical removal can further disrupt biofilm integrity and enhance biocidal activity.

As storage time and temperature increased, samples from both groups shifted toward the negative side of PC1, moving farther away from the landmark of count reduction. This trend, independent of biofilm surface dryness and coupon material, suggests a decline in sanitizer effectiveness over time. By 30 days, most samples from both groups were located near the central axis, while samples stored for 60 days were predominantly found on the negative side of PC1. Older biofilms produce more EPS, and at higher temperatures, metabolic activity and stress responses are enhanced, further reducing susceptibility (Donlan, 2002; Simões et al., 2006).

The negative side of PC2, dominated by biofilm mechanical removal, reflects the reduced efficacy of sanitizers on unscrubbed surfaces, as scrubbing disrupts the EPS matrix and enhances sanitizer penetration into biofilms and contact with attached cells. Others have also found that mechanical cleaning significantly reduces bacterial numbers and prevents biofilm-associated resistance (Boyce, 2016).

The PC1 × PC3 plot (Figure 1B) revealed a distinct separation between SS, positioned on the positive side of PC3, and TPU coupons, located on the negative side, suggesting the sanitizer efficacy was impacted by surface material. In both material groups, most samples stored for 6 days at either 10 °C or 25 °C, regardless of mechanical removal or biofilm surface moisture, were positioned on the positive side of PC1 and closely aligned with the count reduction vector, suggesting that the organic peroxy acid sanitizer (S1 = PAA) was effective at reducing O157:H7 populations, particularly in freshly formed biofilms (6 days), irrespective of surface material or mechanical treatment. Notably, the SS group demonstrated a more pronounced reduction than TPU, which can be attributed to the smoother and less hydrophobic nature of SS, which discourages biofilm adherence and facilitates sanitizer penetration (Sinde and Carballo, 2000; Donlan and Costerton, 2002). As storage time (biofilm age) and temperature increased, samples from both material types progressively shifted toward the negative side of PC1 and away from the count reduction vector, reflecting decreased sanitizer efficacy. This observation is consistent with the structural maturation of biofilms and enhanced EPS production, which impede sanitizer access to embedded bacteria (Xue et al., 2012; Hua et al., 2019a).

**Figure 2:**
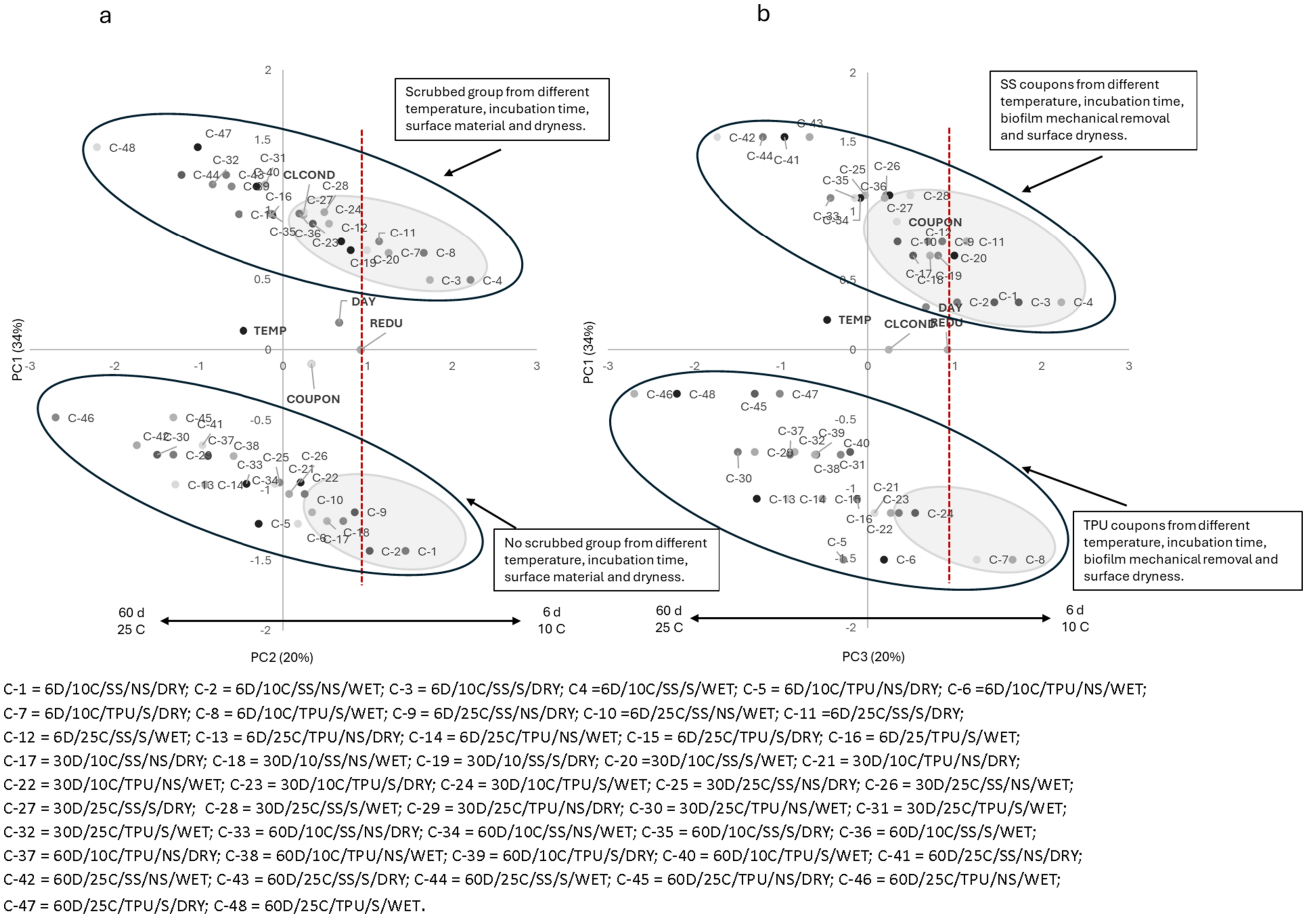
**(a)** Plotting principal component (PC1 × PC2) analysis for biofilm pathogen reduction (REDU) under different environment and management conditions when *quaternary ammonium compounds (GM)* was applied. **(b)** Plotting principal component (PC1 × PC3) analysis for biofilm pathogen reduction under different environments and management conditions when GM was applied. Gray-shaded regions indicate sample groupings, and the red vertical line represents the landmark for count reduction. Factors positioned on the positive side of PC1 and near or to the right of the landmark suggest *E. coli* 0157: H7 susceptibility to GM. Storing day (DAY): 6 = 6D, 30 = 30D, 60 = 60D days. Temperature (TEMP): 10 °C vs. 25 °C. Coupon material (COUPON): stainless steel = SS, polyurethane = TPU. Biofilm mechanical removal (BMR): scrubbed = S, no scrubbed = NS.

**Figure 3:**
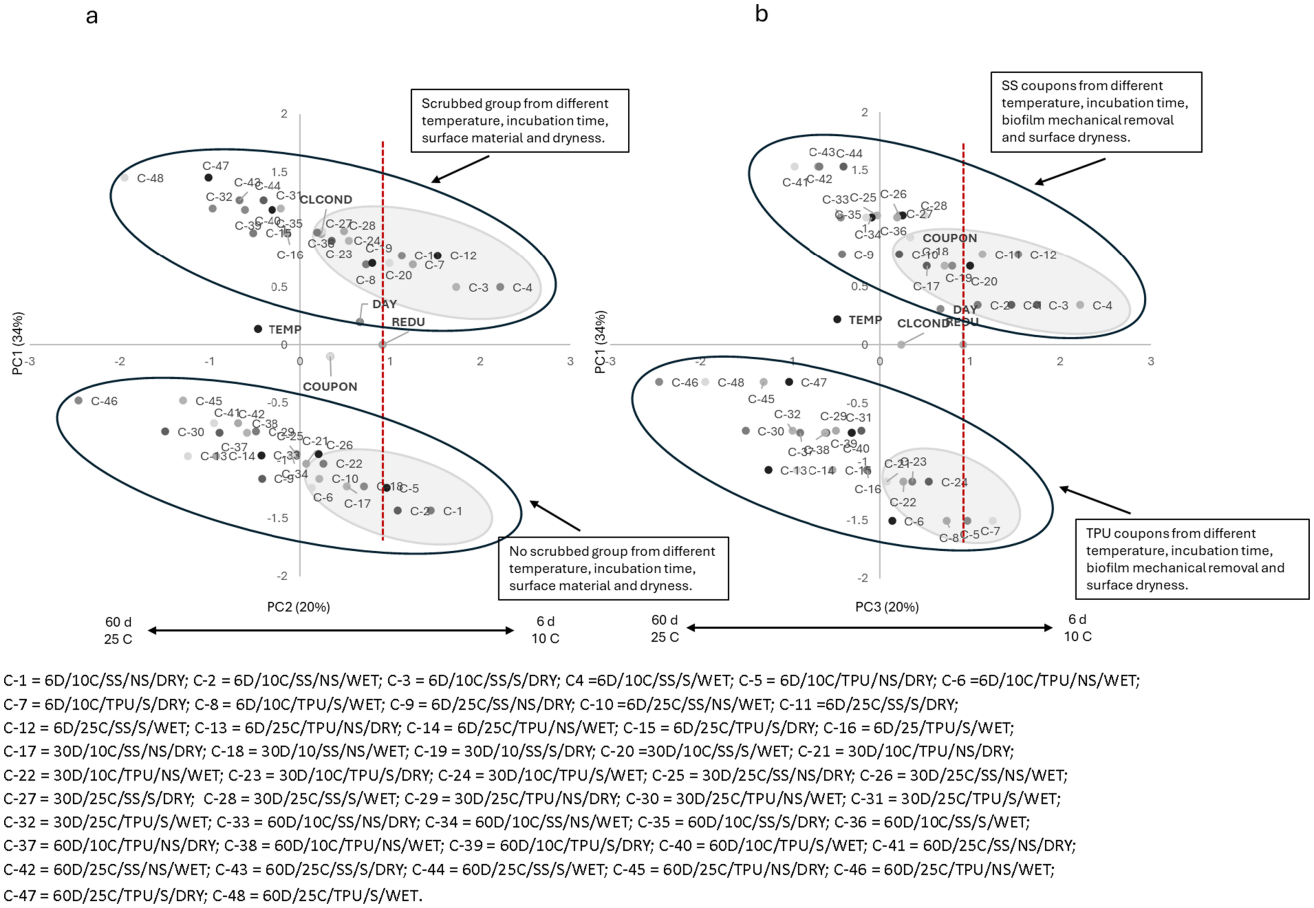
**(a)** Plotting principal component (PC1 × PC2) analysis for biofilm pathogen reduction (REDU) under different environment and management conditions when *sodium hydroxide (SHyd) was applied*. **(b)** Plotting principal component (PC1 × PC3) analysis for biofilm pathogen reduction under different environments and management conditions when SHyd was applied. Grey-shaded regions indicate sample groupings, and the red vertical line represents the landmark for count reduction. Factors positioned on the positive side of PC1 and near or to the right of the landmark suggest *E. coli* 0157: H7 susceptibility to SHyd. Storing day (DAY): 6 = 6D, 30 = 30D, 60 = 60D days. Temperature (TEMP): 10 °C vs. 25 °C. Coupon material (COUPON): stainless steel = SS, polyurethane = TPU. Biofilm mechanical removal (BMR): scrubbed = S, no scrubbed = NS.

**Figure 4:**
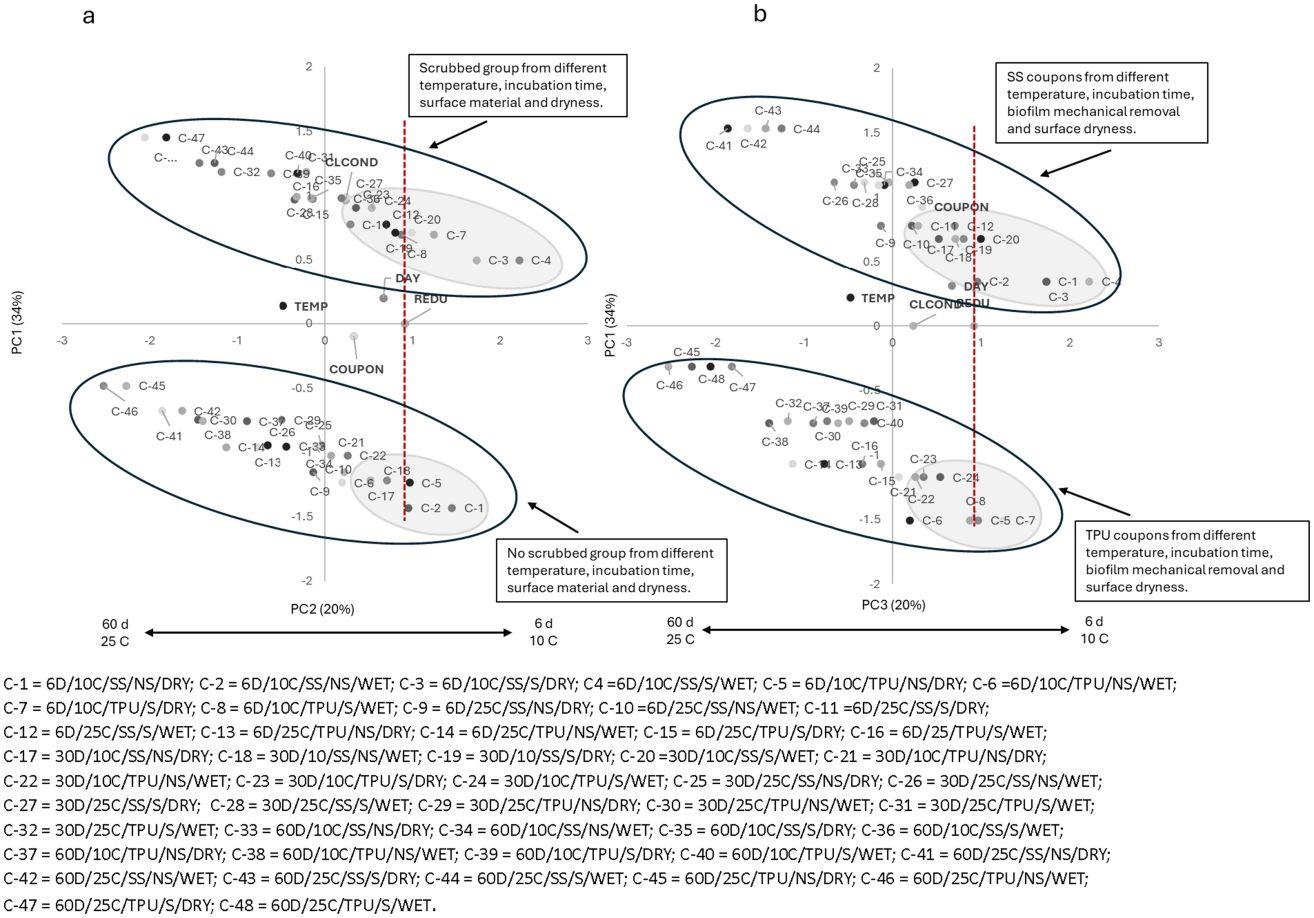
**(a)** Plotting principal component (PC1 × PC2) analysis for biofilm pathogen reduction (REDU) under different environment and management conditions when *hydrogen peroxide (HyP)* was applied. **(b)** Plotting principal component (PC1 × PC3) analysis for biofilm pathogen reduction under different environments and management conditions when HyP was applied. Gray-shaded regions indicate sample groupings, and the red vertical line represents the landmark for count reduction. Factors positioned on the positive side of PC1 and near or to the right of the landmark suggest *E. coli* 157: H7 susceptibility to HyP. Storing day (DAY): 6 = 6D, 30 = 30D, 60 = 60D days. Temperature (TEMP): 10 °C vs. 25 °C. Coupon material (COUPON): stainless steel = SS, polyurethane = TPU. Biofilm mechanical removal (BMR): scrubbed = S, no scrubbed = NS.

**Figure 5:**
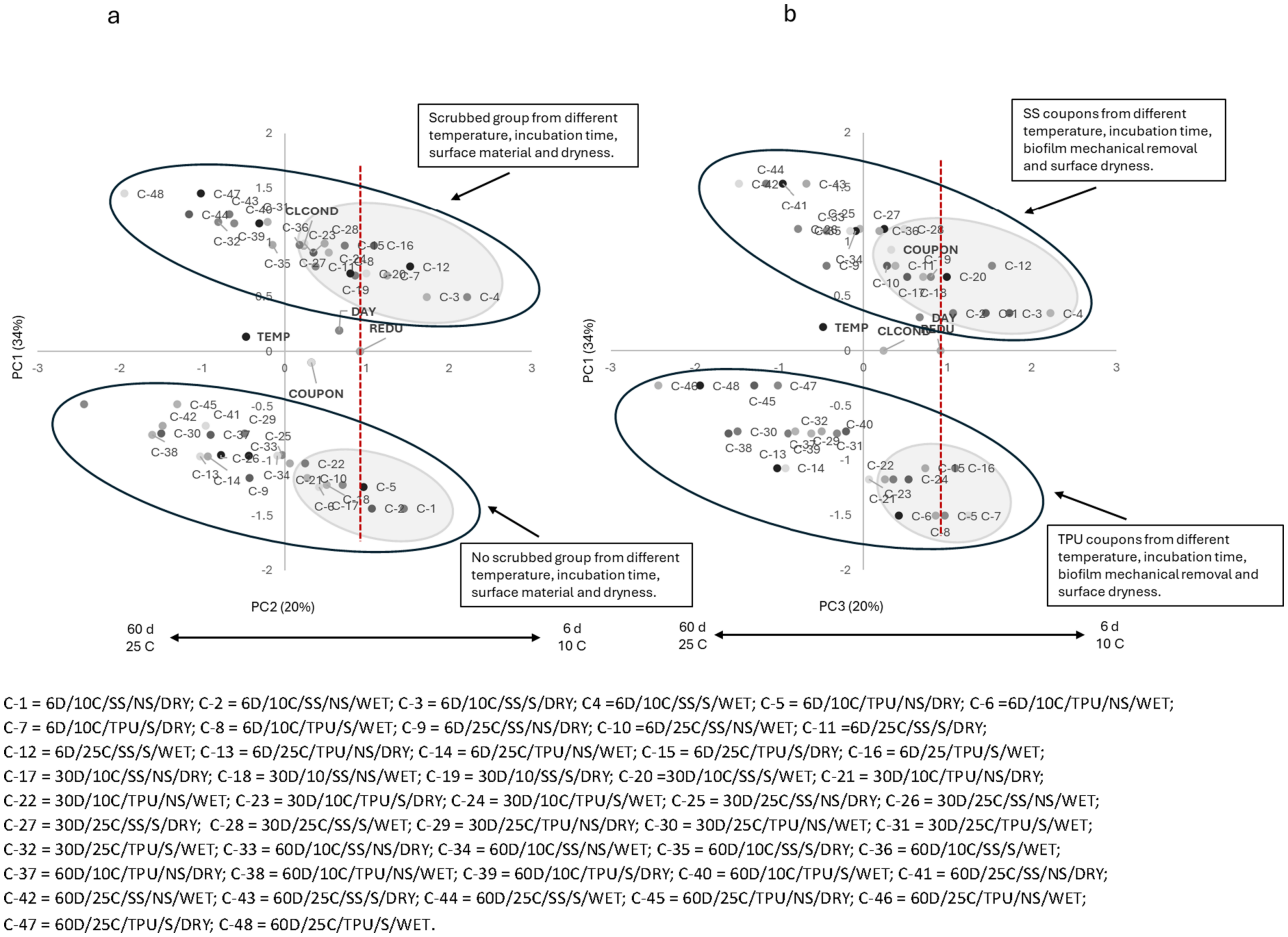
**(a)** Plotting principal component (PC1 × PC2) analysis for biofilm pathogen reduction (REDU) under different environment and management conditions when *quaternary ammonium compounds (PQ)* was applied. **(b)** Plotting principal component (PC1 × PC3) analysis for biofilm pathogen reduction under different environments and management conditions when *PQ* was applied. Gray-shaded regions indicate sample groupings, and the red vertical line represents the landmark for count reduction. Factors positioned on the positive side of PC1 and near or to the right of the landmark suggest *E. coli* O157:H7 susceptibility to PQ. Storing day (DAY): 6 = 6D, 30 = 30D, 60 = 60D days. Temperature (TEMP): 10 °C vs. 25 °C. Coupon material (COUPON): polyurethane = TPU. Biofilm mechanical removal (BMR): scrubbed = S, no scrubbed = NS.

**Figure 6:**
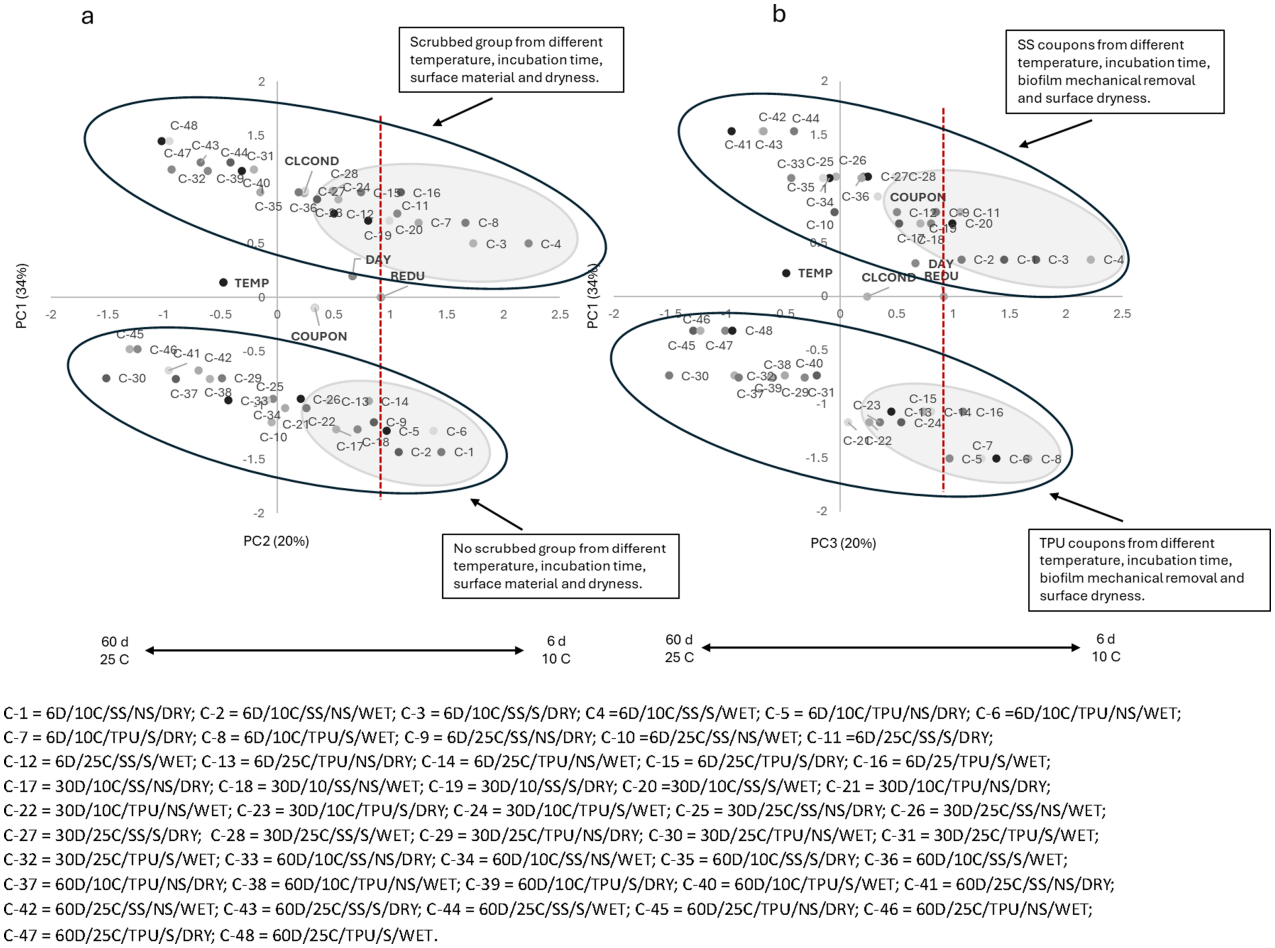
**(a)** Plotting principal component (PC1 × PC2) analysis for biofilm pathogen reduction (REDU) under different environment and management conditions when *sodium hypochlorite (Shypo)* was applied. **(b)** Plotting principal component (PC1 × PC3) analysis for biofilm pathogen reduction under different environments and management conditions when Shypo was applied. Gray-shaded regions indicate sample groupings, and the red vertical line represents the landmark for count reduction. Factors positioned on the positive side of PC1 and near or to the right of the landmark suggest *E. coli* 0157: H7 susceptibility to Shypo. Storing day (DAY): 6 = 6D, 30 = 30D, 60 = 60D days. Temperature (TEMP): 10 °C vs. 25 °C. Coupon material (COUPON): polyurethane = TPU. Biofilm mechanical removal (BMR): scrubbed = S, no scrubbed = NS.

Similar trends were observed for all tested sanitizers (Figures 2–6), where higher pathogen reduction consistently occurred on scrubbed SS surfaces stored for 6 days at 10 °C, regardless of surface moisture, highlighting shared relationships among all sanitizers with respect to mechanical removal and surface material.

The PC analysis revealed that specific combinations of environmental and management factors, such as storage temperature, biofilm age, mechanical removal, and surface type, consistently influenced the effectiveness of each sanitizer in reducing bacterial counts.

### 3.2 Sanitizer effectiveness comparison in similar combined conditions on single-species and spoilage multispecies biofilms

The sanitizers were compared specifically at 6 d of incubation, at 10 °C and 25 °C, on both TPU and SS surfaces, with and without mechanical scrubbing, under both wet and dry conditions.

#### 3.2.1 Single-species O157 biofilms

**Figure 7:**
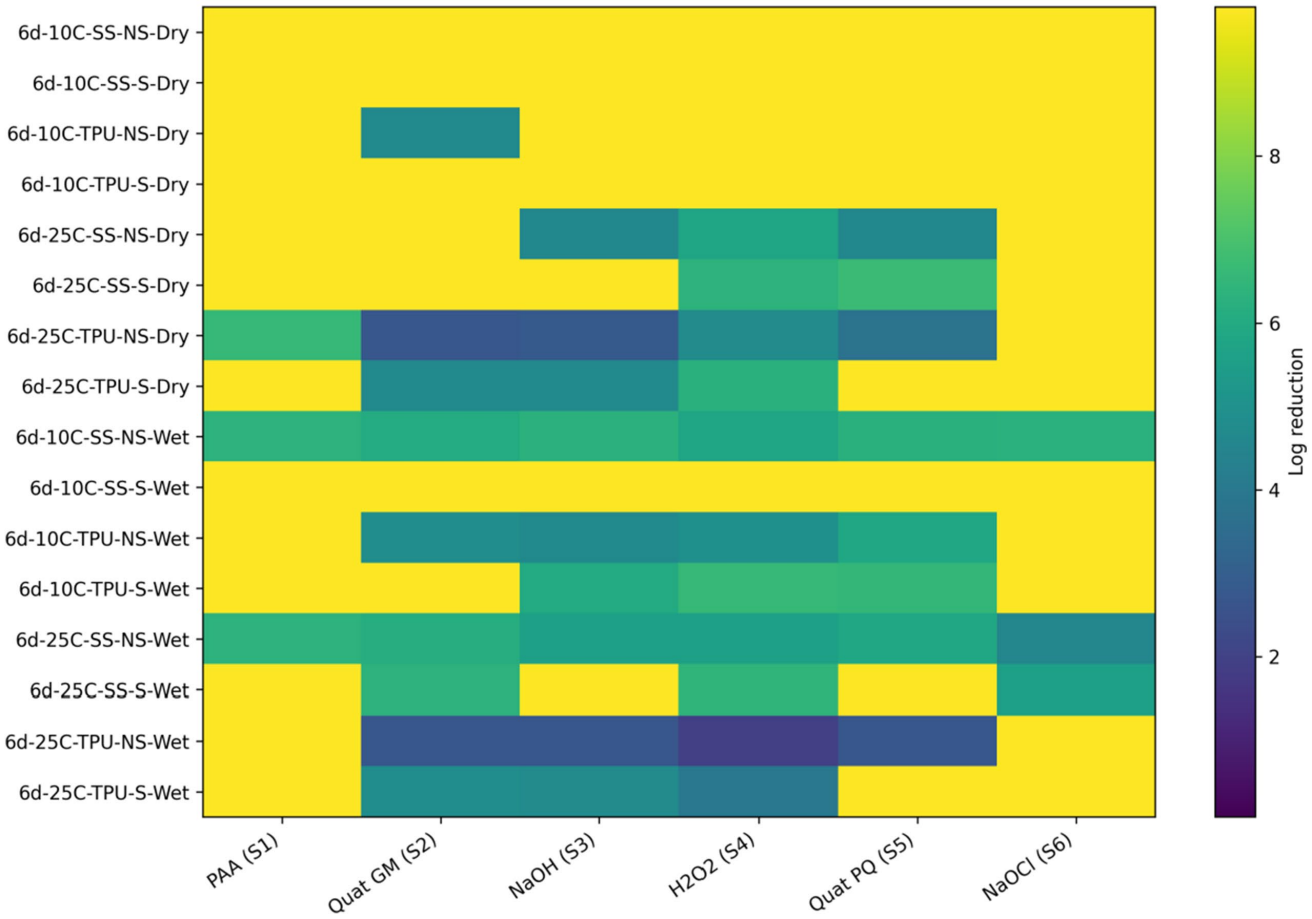
Heat map of sanitizer efficacy (log reduction) against single-species *E. coli* 0157 biofilms. Sanitizers: S1 = peroxyacetic acid (PAA, BioDestroy, 600 ppm); S2 = quaternary ammonium compound (GM, 600 ppm); S3 = sodium hydroxide (2,500 ppm); S4 = hydrogen peroxide (250 ppm); S5 = quaternary ammonium compound (PQ, 550 ppm); S6 = sodium hypochlorite (1,200 ppm). Conditions: 6 d storage; 10 °C or 25 °C; stainless steel (SS) or polyurethane (TPU); scrubbed (S) or non-scrubbed (NS); dry vs. wet biofilm surface (*p ≤* 0.05). The color gradient indicates the magnitude of log reduction.

Overall, the sanitizers ranked from least to most effective were: hydrogen peroxide < sodium hydroxide < Quats (GM) < Quats (PQ) < sodium hypochlorite < BioDestroy (Figure 7; Table 4). To complement the visual representation shown in the heat maps, the accompanying tables report the quantitative log reductions and the results of statistical analyses comparing treatments within each condition. Visual inspection of the heat map indicates that wet biofilms exhibited greater tolerance to sanitization, as evidenced by the higher frequency of lower log reductions (blue to dark-green color range) compared with dry biofilms, which more frequently displayed higher reductions (yellow to light-green)., this findings could be attributed to several factors. The high-water content in wet biofilms supports bacterial metabolic activity and osmotic balance, reducing the efficacy of sanitizers that rely on osmotic disruption (Bridier et al., 2011). Additionally, wet biofilms often contain higher levels of organic and inorganic materials, which can react with sanitizers such as sodium hypochlorite or hydrogen peroxide, depleting their active components before bacterial contact (Boyce, 2016). Structurally, wet biofilms, though less compact, possess cohesive properties that shield against sanitizer penetration (Donlan, 2002). Regarding temperature, biofilms at 10 °C were more susceptible than biofilms formed at 25 °C. This increased resilience at 25 °C may be due to more EPS biomass, but also to the expression of stress-resistance genes by *E. coli* O157, including heat shock proteins and oxidative stress enzymes, which enhance tolerance to oxidative damage (Fee, 1991; Karavolos et al., 2003). The hydrophobic nature of TPU surfaces further interacts with water-based sanitizers, reducing their adhesion and efficacy compared to SS surfaces, which were associated with more effective pathogen reductions (Mah and O’Toole, 2001). Lastly, non-scrubbed biofilms showed lesser reductions in *E. coli* O157, indicating the critical role of mechanical removal in enhancing sanitizer performance. Scrubbing effectively disrupts biofilm integrity, removing attached cells and reducing the protective matrix that shields pathogens from sanitizer action (Van Houdt and Michiels, 2010).

**Table 4:** Effect of the different sanitizers^1^ single species *E. coli* O157 biofilm formed under different environmental and management conditions (*p* < 0.0001; SEM = 0.03).

Condition	Biofilm surface dryness	S1	S2	S3	S4	S5	S6
6d-10C-SS-NS	Dry	9.77 ^A/a/x^	9.77 ^A/a/x^	9.77 ^A/a/x^	9.77 ^A/a/x^	9.77 ^A/a/x^	9.77 ^A/a/x^
6d-10C-SS-S		9.77 ^A/a/x^	9.77 ^A/a/x^	9.77 ^A/a/x^	9.77 ^A/a/x^	9.77 ^A/a/x^	9.77 ^A/a/x^
6d-10C-TPU-NS		9.77 ^A/a/x^	4.69 ^B/b/x^	9.77 ^A/a/x^	9.77 ^A/a/x^	9.77 ^A/a/x^	9.77 ^A/a/x^
6d-10C-TPU-S		9.77 ^A/a/x^	9.77 ^A/a/x^	9.77 ^A/a/x^	9.77 ^A/a/x^	9.77 ^A/a/x^	9.77 ^A/a/x^
6d-25C-SS-NS		9.77 ^A/a/x^	9.77 ^A/a/x^	4.59 ^C/b/x^	5.79 ^B/d/x^	4.57 ^C/c/x^	9.77 ^A/a/x^
6d-25C-SS-S		9.77 ^A/a/x^	9.77 ^A/a/x^	9.77 ^A/a/x^	6.35 ^C/b/x^	6.71 ^B/b/x^	9.77 ^A/a/x^
6d-25C-TPU-NS		6.58 ^B/b/x^	2.72 ^E/c/x^	2.85 ^E/c/x^	4.76 ^C/e/x^	3.76 ^D/d/x^	9.77 ^A/a/x^
6d-25C-TPU-S		9.77 ^A/a/x^	4.64 ^C/b/x^	4.64 ^C/b/x^	6.24 ^B/c/x^	9.77 ^A/a/x^	9.77 ^A/a/x^
6d-10C-SS-NS	Wet	6.37 ^A/b/y^	6.07 ^C/c/y^	6.32 ^A/b/y^	5.81 ^D/d/y^	6.26 ^B/c/y^	6.27 ^B/b/y^
6d-10C-SS-S		9.79 ^A/a/x^	9.79 ^A/a/x^	9.79 ^A/a/x^	9.79 ^A/a/x^	9.79 ^A/a/x^	9.79 ^A/a/x^
6d-10C-TPU-NS		9.78 ^A/a/x^	4.90 ^C/d/y^	4.71 ^D/e/y^	4.97 ^C/f/y^	5.88 ^B/d/y^	9.78 ^A/a/x^
6d-10C-TPU-S		9.78 ^A/a/x^	9.78 ^A/a/x^	6.01 ^C/c/y^	6.57 ^B/b/y^	6.51 ^B/b/y^	9.78 ^A/a/x^
6d-25C-SS-NS		6.35 ^A/b/y^	6.12 ^B/c/y^	5.59 ^D/d/y^	5.62 ^D/e/y^	5.86 ^C/d/y^	4.53 ^E/d/y^
6d-25C-SS-S		9.78 ^A/a/x^	6.37 ^B/b/x^	9.78 ^A/a/x^	6.43 ^B/c/y^	9.78 ^A/a/y^	5.62 ^C/c/y^
6d-25C-TPU-NS		9.78 ^A/a/x^	2.74 ^B/e/x^	2.71 ^B/f/y^	1.91 ^C/h/y^	2.73 ^B/e/y^	9.78 ^A/a/x^
6d-25C-TPU-S		9.78 ^A/a/x^	4.85 ^B/d/x^	4.77 ^C/e/y^	3.98 ^D/g/y^	9.78 ^A/a/x^	9.78 ^A/a/x^

^1^Sanitizers: S1 = PAA (Organic peroxy acids or BioDestroy^®^) 600 ppm; S2 = Quats (GM) 600 ppm; S3 = Sodium hydroxide (SHyd) 2,500 ppm; S4 = Hydrogen peroxide (HyP) 250 ppm; S5 = Quats (PQ) = 550 ppm; S6 = Sodium hypochlorite (Shypo) 1,200 ppm. Storing day: 6 days = 6d. Temperature: 10 °C vs. 25 °C. Coupon material: stainless steel = SS, polyurethane = TPU. Biofilm mechanical removal: scrubbed = S, no scrubbed = NS. Controls: Biofilms non-treated with the biocides. A-F/ = indicates significant differences (*p ≤* 0.05) between sanitizers within each environment and management condition combination and biofilm surface dryness. /a-h/= indicates differences (*p ≤* 0.05) between combined conditions for the same biofilm surface dryness and sanitizer. /xy = indicates differences (*p ≤* 0.05) between biofilm surface dryness for the same combined condition and sanitizer.

#### 3.2.2 Multispecies biofilm combinations

**Figure 8:**
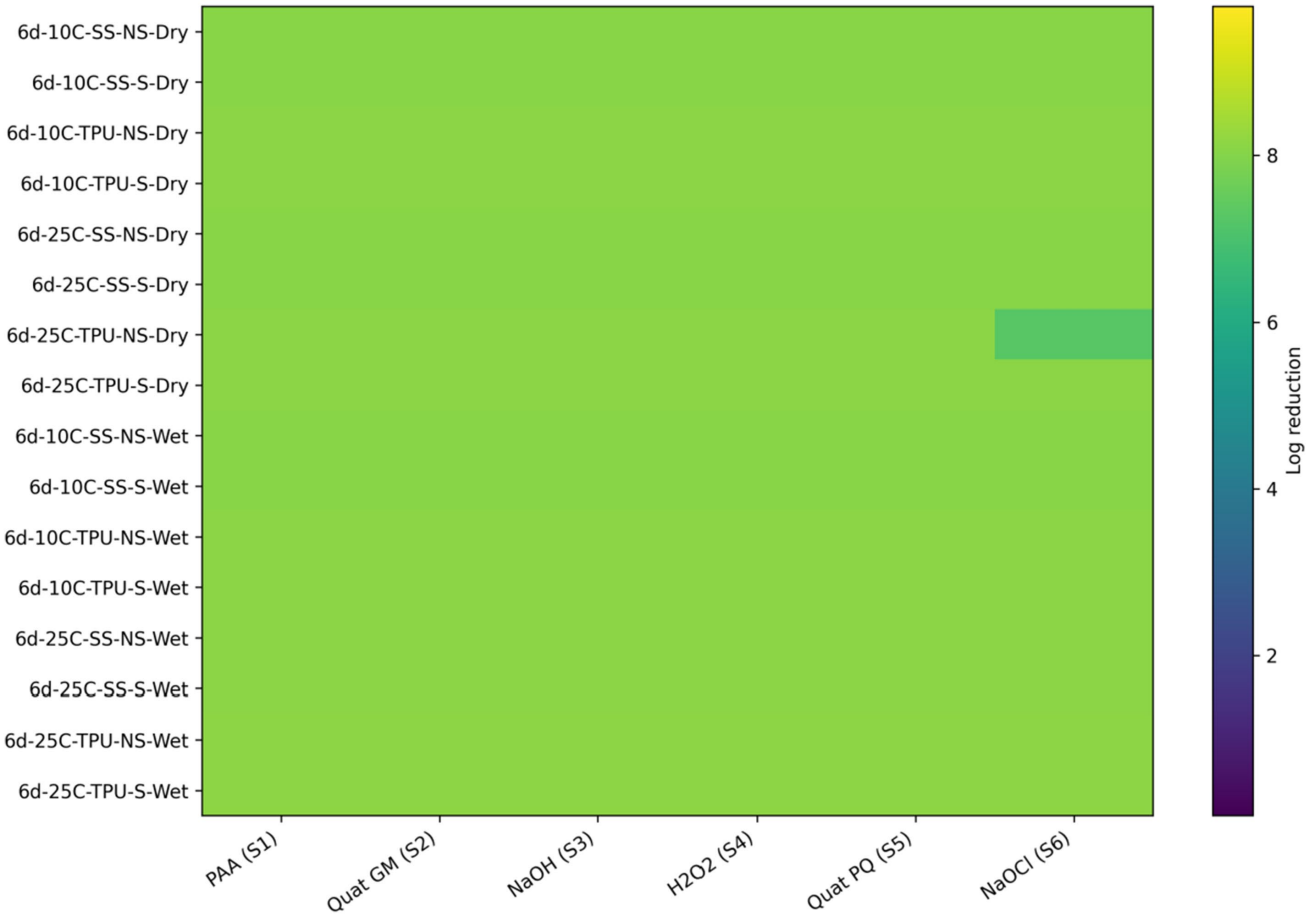
Heat map of sanitizer efficacy (log reduction) against multispecies biofilms (T1) - spoilage bacteria. Sanitizers: S1 = peroxyacetic acid (PAA, BioDestroy^®^, 600 ppm); S2 = quaternary ammonium compound (GM, 600 ppm); S3 = sodium hydroxide (2,500 ppm); S4 = hydrogen peroxide (250 ppm); S5 = quaternary ammonium compound (PQ, 550 ppm); S6 = sodium hypochlorite (1,200 ppm). Conditions: 6 d storage; 10 °C or 25 °C; stainless steel (SS) or polyurethane (TPU); scrubbed (S) or non-scrubbed (NS); dry vs. wet biofilm surface (*p ≤* 0.05). The color gradient indicates the magnitude of log reduction.

All sanitizers were highly effective in reducing LAB bacteria; however, only one sanitizer, sodium hypochlorite, failed to completely eliminate LAB bacteria *Carnobacterium* and *Lactobacillus* from biofilms on non-scrubbed TPU surfaces at 25 °C (Figure 8; Table 5).

**Table 5:** Effect of the different sanitizers^1^ applied to multiple pathogen species biofilm formed under different environmental and management conditions, and reduction values are noted for spoilage bacteria (T1) (*p <* 0.0001; SEM = 0.06).

Condition	Biofilm surface dryness	S1	S2	S3	S4	S5	S6
6d-10C-SS-NS	Dry	8.07 ^A/a/x^	8.07 ^A/a/x^	8.07 ^A/a/x^	8.07 ^A/a/x^	8.07 ^A/a/x^	8.07 ^A/a/x^
6d-10C-SS-S		8.07 ^A/a/x^	8.07 ^A/a/x^	8.07 ^A/a/x^	8.07 ^A/a/x^	8.07 ^A/a/x^	8.07 ^A/a/x^
6d-10C-TPU-NS		8.09 ^A/a/x^	8.09 ^A/a/x^	8.09 ^A/a/x^	8.09 ^A/a/x^	8.09 ^A/a/x^	8.09 ^A/a/x^
6d-10C-TPU-S		8.09 ^A/a/x^	8.09 ^A/a/x^	8.09 ^A/a/x^	8.09 ^A/a/x^	8.09 ^A/a/x^	8.09 ^A/a/x^
6d-25C-SS-NS		8.08 ^A/a/x^	8.08 ^A/a/x^	8.08 ^A/a/x^	8.08 ^A/a/x^	8.08 ^A/a/x^	8.08 ^A/a/x^
6d-25C-SS-S		8.08 ^A/a/x^	8.08 ^A/a/x^	8.08 ^A/a/x^	8.08 ^A/a/x^	8.08 ^A/a/x^	8.08 ^A/a/x^
6d-25C-TPU-NS		8.11 ^A/a/x^	8.11 ^A/a/x^	8.11 ^A/a/x^	8.11 ^A/a/x^	8.11 ^A/a/x^	7.28 ^B/b/x^
6d-25C-TPU-S		8.11 ^A/a/x^	8.11 ^A/a/x^	8.11 ^A/a/x^	8.11 ^A/a/x^	8.11 ^A/a/x^	8.11 ^A/a/x^
6d-10C-SS-NS	Wet	8.08 ^A/a/x^	8.08 ^A/a/x^	8.08 ^A/a/x^	8.08 ^A/a/x^	8.08 ^A/a/x^	8.08 ^A/a/x^
6d-10C-SS-S		8.08 ^A/a/x^	8.08 ^A/a/x^	8.08 ^A/a/x^	8.08 ^A/a/x^	8.08 ^A/a/x^	8.08 ^A/a/x^
6d-10C-TPU-NS		8.10 ^A/a/x^	8.10 ^A/a/x^	8.10 ^A/a/x^	8.10 ^A/a/x^	8.10 ^A/a/x^	8.10 ^A/a/x^
6d-10C-TPU-S		8.10 ^A/a/x^	8.10 ^A/a/x^	8.10 ^A/a/x^	8.10 ^A/a/x^	8.10 ^A/a/x^	8.10 ^A/a/x^
6d-25C-SS-NS		8.09 ^A/a/x^	8.09 ^A/a/x^	8.09 ^A/a/x^	8.09 ^A/a/x^	8.09 ^A/a/x^	8.09 ^A/a/x^
6d-25C-SS-S		8.09 ^A/a/x^	8.09 ^A/a/x^	8.09 ^A/a/x^	8.09 ^A/a/x^	8.09 ^A/a/x^	8.09 ^A/a/x^
6d-25C-TPU-NS		8.16 ^A/a/x^	8.16 ^A/a/x^	8.16 ^A/a/x^	8.16 ^A/a/x^	8.16 ^A/a/x^	8.16 ^A/a/x^
6d-25C-TPU-S		8.16 ^A/a/x^	8.16 ^A/a/x^	8.16 ^A/a/x^	8.16 ^A/a/x^	8.16 ^A/a/x^	8.16 ^A/a/x^

^1^Sanitizers: S1 = PAA (Organic peroxy acids or BioDestroy^®^) 600 ppm; S2 = Quats (GM) 600 ppm; S3 = Sodium hydroxide (SHyd) 2,500 ppm; S4 = Hydrogen peroxide (HyP) 250 ppm; S5 = Quats (PQ) = 550 ppm; S6 = Sodium hypochlorite (Shypo) 1,200 ppm. Storing day: 6 days = 6d. Temperature: 10 °C vs. 25 °C. Coupon material: stainless steel = SS, polyurethane = TPU. Biofilm mechanical removal: scrubbed = S, no scrubbed = NS. Controls: Biofilms non-treated with the biocides A-F/ = indicates significant differences (*p ≤* 0.05) between sanitizers within each environment and management condition combination and biofilm surface dryness. /a-g/= indicates differences (*p ≤* 0.05) between combined conditions for the same biofilm surface dryness and sanitizer. /xy = indicates differences (*p ≤* 0.05) between biofilm surface dryness for the same combined condition and sanitizer.

**Figure 9:**
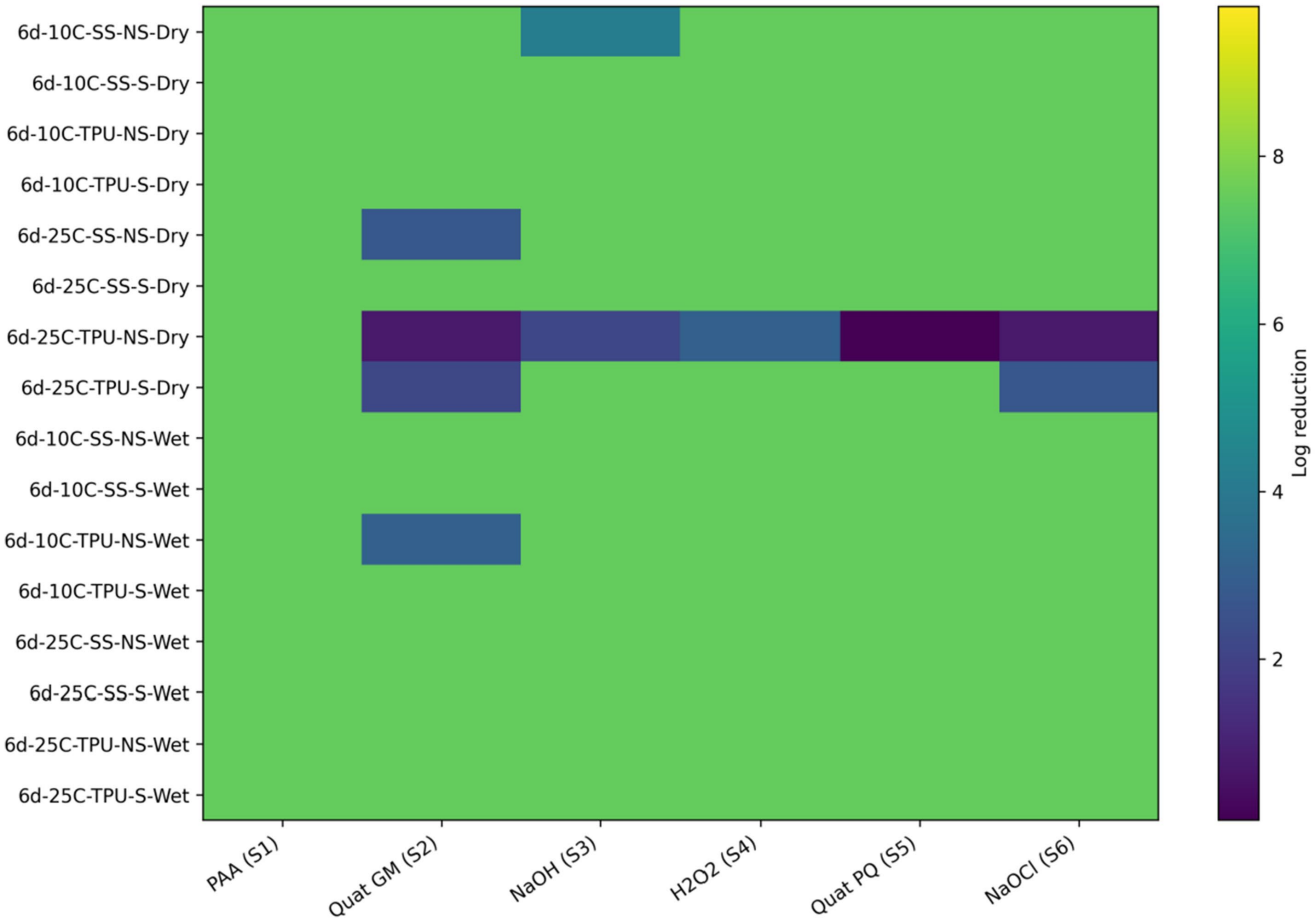
Heat map of sanitizer efficacy (log reduction) against multispecies biofilms (T1) - *E. coli* 0157: H7. Sanitizers: S1 = peroxyacetic acid (PAA, BioDestroy^®^, 600 ppm); S2 = quaternary ammonium compound (GM, 600 ppm); S3 = sodium hydroxide (2,500 ppm); S4 = hydrogen peroxide (250 ppm); S5 = quaternary ammonium compound (PQ, 550 ppm); S6 = sodium hypochlorite (1,200 ppm). Conditions: 6 d storage; 10 °C or 25 °C; stainless steel (SS) or polyurethane (TPU); scrubbed (S) or non-scrubbed (NS); dry vs. wet biofilm surface (*p ≤* 0.05). The color gradient indicates the magnitude of log reduction.

When examining O157:H7, a distinct pattern emerged for 6-day dry biofilms formed at 25 °C on unscrubbed TPU surfaces. Under these conditions, most sanitizers failed to achieve complete inactivation, as reflected by the predominance of lower log reductions (blue to purple regions) across treatments in the heat map (Figure 9; Table 6). This pattern contrasts with the higher reductions observed under scrubbed conditions or on stainless steel surfaces, indicating that the combination of environmental temperature, dry biofilm formation, lack of mechanical removal, and TPU surface material represents a particularly challenging sanitation scenario to eliminate O157:H7. The only sanitizer that was effective in these conditions was Biodestroy (PAA), which promotes biofilm disruption and penetration and exhibits broad-spectrum biocidal activity (Donlan, 2002; Koti et al., 2024). When looking at the other environmental and treatment conditions, it was also found that the biocidal activity of Quats (GM) was reduced on both unscrubbed SS (2.75 log₁₀ CFU/cm^2^ reduction) and scrubbed TPU surfaces (2.21 log₁₀ CFU/cm^2^ reduction). This reduced efficacy may be attributed to different surface characteristics, lower surface energy and higher hydrophobicity in TPU, and reduced mechanical disruption on unscrubbed SS, both of which can limit sanitizer access and penetration. Sodium hypochlorite also exhibited reduced activity (4.19 log_10_ CFU/cm^2^ reductions) on non-scrubbed SS surfaces. The effectiveness of sodium hypochlorite was also reduced on scrubbed TPU surfaces (2.71 log_10_ °CFU/cm^2^ reductions). The absence of mechanical removal likely left residual biofilm layers that were partially inactivated and/or impeded sanitizer access to bacterial cells embedded deeper in biofilms (Boyce, 2016).

**Table 6:** Effect of the different sanitizers^1^ applied to multiple pathogen species biofilm formed under different environmental and management conditions, and reduction values are noted for *E. coli* O157:H7 (T1) (*p <* 0.0001; SEM = 0.04).

Condition	Biofilm surface dryness	S1	S2	S3	S4	S5	S6
6d-10C-SS-NS	Dry	7.49 ^A/a/x^	7.49 ^A/a/x^	4.19 ^B/b/x^	7.49 ^A/a/x^	7.49 ^A/a/x^	7.49 ^A/a/x^
6d-10C-SS-S		7.49 ^A/a/x^	7.49 ^A/a/x^	7.49 ^A/a/x^	7.49 ^A/a/x^	7.49 ^A/a/x^	7.49 ^A/a/x^
6d-10C-TPU-NS		7.49 ^A/a/x^	7.49 ^A/a/x^	7.49 ^A/a/x^	7.49 ^A/a/x^	7.49 ^A/a/x^	7.49 ^A/a/x^
6d-10C-TPU-S		7.49 ^A/a/x^	7.49 ^A/a/x^	7.49 ^A/a/x^	7.49 ^A/a/x^	7.49 ^A/a/x^	7.49 ^A/a/x^
6d-25C-SS-NS		7.50 ^A/a/x^	2.75 ^B/b/x^	7.50 ^A/a/x^	7.50 ^A/a/x^	7.50 ^A/a/x^	7.50 ^A/a/x^
6d-25C-SS-S		7.50 ^A/a/x^	7.50 ^A/a/x^	7.50 ^A/a/x^	7.50 ^A/a/x^	7.50 ^A/a/x^	7.50 ^A/a/x^
6d-25C-TPU-NS		7.50 ^A/a/x^	0.74 ^D/d/x^	2.14 C^/c/x^	3.12 ^B/b/x^	0.09 ^E/b/x^	0.77 ^D/c/x^
6d-25C-TPU-S		7.50 ^A/a/x^	2.21 C^/c/x^	7.51 ^A/a/x^	7.51 ^A/a/x^	7.51 ^A/a/x^	2.71 ^B/b/x^
6d-10C-SS-NS	Wet	7.50 ^A/a/x^	7.50 ^A/a/x^	7.50 ^A/a/y^	7.50 ^A/a/x^	7.50 ^A/a/x^	7.50 ^A/a/x^
6d-10C-SS-S		7.50 ^A/a/x^	7.50 ^A/a/x^	7.50 ^A/a/x^	7.50 ^A/a/x^	7.50 ^A/a/x^	7.50 ^A/a/x^
6d-10C-TPU-NS		7.50 ^A/a/x^	3.11 ^B/b/y^	7.50 ^A/a/x^	7.50 ^A/a/x^	7.50 ^A/a/x^	7.50 ^A/a/x^
6d-10C-TPU-S		7.50 ^A/a/x^	7.50 ^A/a/x^	7.50 ^A/a/x^	7.50 ^A/a/x^	7.50 ^A/a/x^	7.50 ^A/a/x^
6d-25C-SS-NS		7.50 ^A/a/x^	7.50 ^A/a/x^	7.50 ^A/a/x^	7.50 ^A/a/x^	7.50 ^A/a/x^	7.50 ^A/a/x^
6d-25C-SS-S		7.50 ^A/a/x^	7.50 ^A/a/x^	7.50 ^A/a/x^	7.50 ^A/a/x^	7.50 ^A/a/x^	7.50 ^A/a/x^
6d-25C-TPU-NS		7.51 ^A/a/x^	7.51 ^A/a/y^	7.51 ^A/a/y^	7.51 ^A/a/y^	7.51 ^A/a/y^	7.51 ^A/a/y^
6d-25C-TPU-S		7.51 ^A/a/x^	7.51 ^A/a/y^	7.51 ^A/a/x^	7.51 ^A/a/x^	7.51 ^A/a/x^	7.51 ^A/a/y^

^1^Sanitizers: S1 = PAA (Organic peroxy acids or BioDestroy^®^) 600 ppm; S2 = Quats (GM) 600 ppm; S3 = Sodium hydroxide (SHyd) 2,500 ppm; S4 = Hydrogen peroxide (HyP) 250 ppm; S5 = Quats (PQ) = 550 ppm; S6 = Sodium hypochlorite (Shypo) 1,200 ppm. Storing day: 6 days = 6d. Temperature: 10°C vs. 25 °C. Coupon material: stainless steel = SS, polyurethane = TPU. Biofilm mechanical removal: scrubbed = S, no scrubbed = NS. Controls: Biofilms non-treated with the biocides. A-F/ = indicates significant differences (*P ≤* 0.05) between sanitizers within each environment and management condition combination and biofilm surface dryness. /a-g/= indicates differences (*p ≤* 0.05) between combined conditions for the same biofilm surface dryness and sanitizer. /xy = indicates differences (*p ≤* 0.05) between biofilm surface dryness for the same combined condition and sanitizer.

Interestingly, the wet biofilm counterpart for T1 combination showed a better response to sanitizers than the dry biofilms, for wet T1 biofilms, all sanitizers were effective at reducing O157, with Quats (GM) being the exception, which only achieved a 3.11 log_10_ °CFU/cm^2^ reduction in biofilms formed at 10 °C on unscrubbed TPU surfaces (Figure 9; Table 6). This better response in wet biofilms highlights the influence of hydration on biofilm matrix permeability, allowing greater sanitizer penetration.

**Figure 10:**
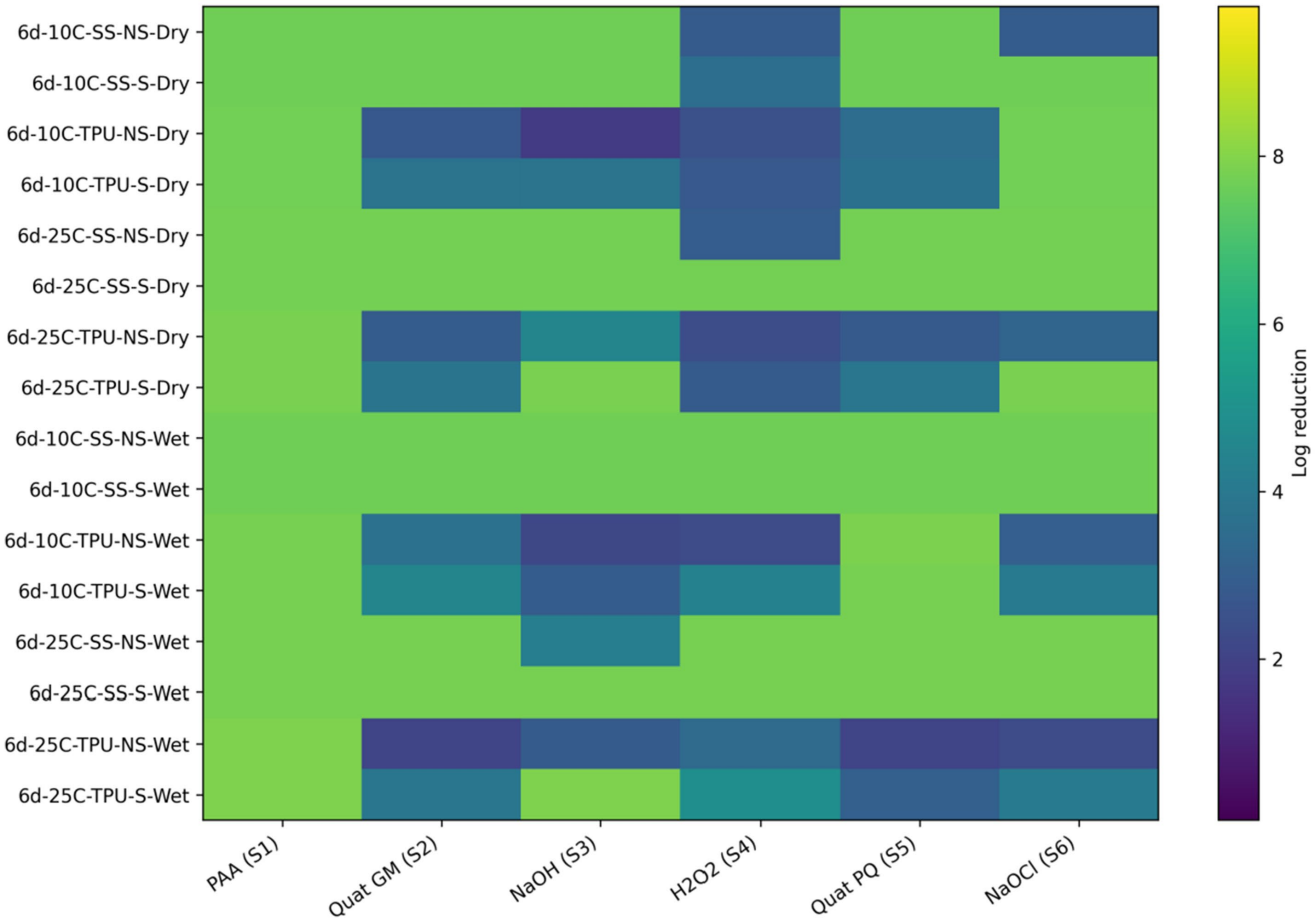
*Heat map* of sanitizer efficacy (log reduction) against multispecies biofilms (T2) - spoilage bacteria. Sanitizers: S1 = peroxyacetic acid (PAA, BioDestroy^®^, 600 ppm); S2 = quaternary ammonium compound (GM, 600 ppm); S3 = sodium hydroxide (2,500 ppm); S4 = hydrogen peroxide (250 ppm); S5 = quaternary ammonium compound (PQ, 550 ppm); S6 = sodium hypochlorite (1,200 ppm). Conditions: 6 d storage; 10 °C or 25 °C; stainless steel (SS) or polyurethane (TPU); scrubbed (S) or non-scrubbed (NS); dry vs. wet biofilm surface (*p ≤* 0.05). The color gradient indicates the magnitude of log reduction.

Sanitizer efficacy against the spoilage bacterial consortium T2: *Comamonas* and *Raoultella* differed markedly from that observed for lactic acid bacteria. Overall, sanitizers were less effective at reducing *Comamonas* and *Raoultella* when compared with LAB (*Carnobacterium* and *Lactobacillus*) as illustrated by the predominance of lower log reductions across conditions in the heat maps (Figure 10; Table 7). Ranking from less effective to more effective sanitizers: hydrogen peroxide < Quats (GM) < sodium hydroxide < Quats (PQ) < sodium hypochlorite < Biodestroy. Biodestroy was effective at reducing SP under all tested conditions. Hydrogen peroxide was less effective on dry biofilms, where reductions ranged from 2.3 to 2.9 (out of 7.7 log_10_ CFU/cm^2^). Similarly, Quats (PQ) were also less effective on dry biofilms. Biofilms formed on TPU surfaces were more resilient (more dark blue and green areas in the heat map) than those on SS. For this bacterial combination, scrubbing did not enhance sanitizer efficacy on TPU, as both scrubbed and non-scrubbed surfaces showed limited reduction, suggesting biofilms on TPU are structurally robust (Figure 10; Table 7).

**Table 7:** Effect of the different sanitizers^1^ applied to multiple pathogen species biofilm formed under different environmental and management conditions, and reduction values are noted for spoilage (T2) (*p <* 0.0001; SEM = 0.11).

Condition	Biofilm surface dryness	S1	S2	S3	S4	S5	S6
6d-10C-SS-NS	Dry	7.67 ^A/a/x^	7.67 ^A/a/x^	7.67 A/a/x	2.86 ^B/b/x^	7.67 ^A/a/x^	2.92 ^A/c/x^
6d-10C-SS-S		7.67 ^A/a/x^	7.67 ^A/a/x^	7.67 A/a/x	3.58 ^B/c/x^	7.67 ^A/a/x^	7.67 ^A/a/x^
6d-10C-TPU-NS		7.74 ^A/a/x^	2.78 C^/a/x^	1.76 E/a/x	2.50 ^D/c/x^	3.52 ^B/c/x^	7.74 ^A/a/x^
6d-10C-TPU-S		7.74 ^A/a/x^	3.80 ^B/c/x^	3.82 B/a/x	2.81 ^D/b/x^	3.66 C^/c/x^	7.74 ^A/a/x^
6d-25C-SS-NS		7.76 ^A/a/x^	7.76 ^A/a/x^	7.76 A/a/x	2.95 ^B/b/x^	7.76 ^A/a/x^	7.75 ^A/a/x^
6d-25C-SS-S		7.76 ^A/a/x^	7.76 ^A/a/x^	7.76 A/a/x	7.76 ^A/a/x^	7.76 ^A/a/x^	7.76 ^A/a/x^
6d-25C-TPU-NS		7.83 ^A/a/x^	2.90 ^D/c/x^	4.49 B/a/x	2.38 ^E/c/x^	2.84 ^D/d/x^	3.22 C^/b/x^
6d-25C-TPU-S		7.83 ^A/a/x^	3.86 ^B/b/x^	7.83 A/a/x	2.86 C^/b/x^	3.91 ^B/b/x^	7.83 ^A/a/x^
6d-10C-SS-NS	Wet	7.69 ^A/a/x^	7.69 ^A/a/x^	7.69 A/a/x	7.69 ^A/a/y^	7.69 ^A/a/x^	7.69 ^A/a/y^
6d-10C-SS-S		7.69 ^A/a/x^	7.69 ^A/a/x^	7.69 A/a/x	7.69 ^A/a/y^	7.69 ^A/a/x^	7.69 ^A/a/x^
6d-10C-TPU-NS		7.79 ^A/a/x^	3.72 ^B/c/y^	2.21 D/a/x	2.34 ^D/a/x^	7.89 ^A/a/y^	3.03 C^/a/y^
6d-10C-TPU-S		7.79 ^A/a/x^	4.50 ^B/b/y^	2.92 D/a/x	4.39 ^B/a/y^	7.79 ^A/a/y^	4.04 C^/a/y^
6d-25C-SS-NS		7.79 ^A/a/x^	7.79 ^A/a/x^	4.24 B/a/x	7.79 ^A/a/y^	7.79 ^A/a/x^	7.79 ^A/a/x^
6d-25C-SS-S		7.79 ^A/a/x^	7.79 ^A/a/x^	7.79 A/a/x	7.79 ^A/a/x^	7.79 ^A/a/x^	7.79 ^A/a/x^
6d-25C-TPU-NS		7.90 ^A/a/x^	2.07 ^E/d/y^	2.86 C/a/x	3.44 ^B/a/y^	2.09 ^E/a/y^	2.35 ^D/a/y^
6d-25C-TPU-S		7.90 ^A/a/x^	3.88 ^D/c/x^	7.90 A/a/x	4.93 ^B/a/y^	3.08 ^E/a/y^	4.12 C^/a/y^

^1^Sanitizers: S1 = PAA (Organic peroxy acids or BioDestroy^®^) 600 ppm; S2 = Quats (GM) 600 ppm; S3 = Sodium hydroxide (SHyd) 2,500 ppm; S4 = Hydrogen peroxide (HyP) 250 ppm; S5 = Quats (PQ) = 550 ppm; S6 = Sodium hypochlorite (Shypo) 1,200 ppm. Storing day: 6 days = 6d. Temperature: 10°C vs. 25 °C. Coupon material: stainless steel = SS, polyurethane = TPU. Biofilm mechanical removal: scrubbed = S, no scrubbed = NS. Controls: Biofilms non-treated with the biocides. A-F/ = indicates significant differences (*P ≤* 0.05) between sanitizers within each environment and management condition combination and biofilm surface dryness. /a-g/= indicates differences (*p ≤* 0.05) between combined conditions for the same biofilm surface dryness and sanitizer. /xy = indicates differences (*p ≤* 0.05) between biofilm surface dryness for the same combined condition and sanitizer.

**Figure 11:**
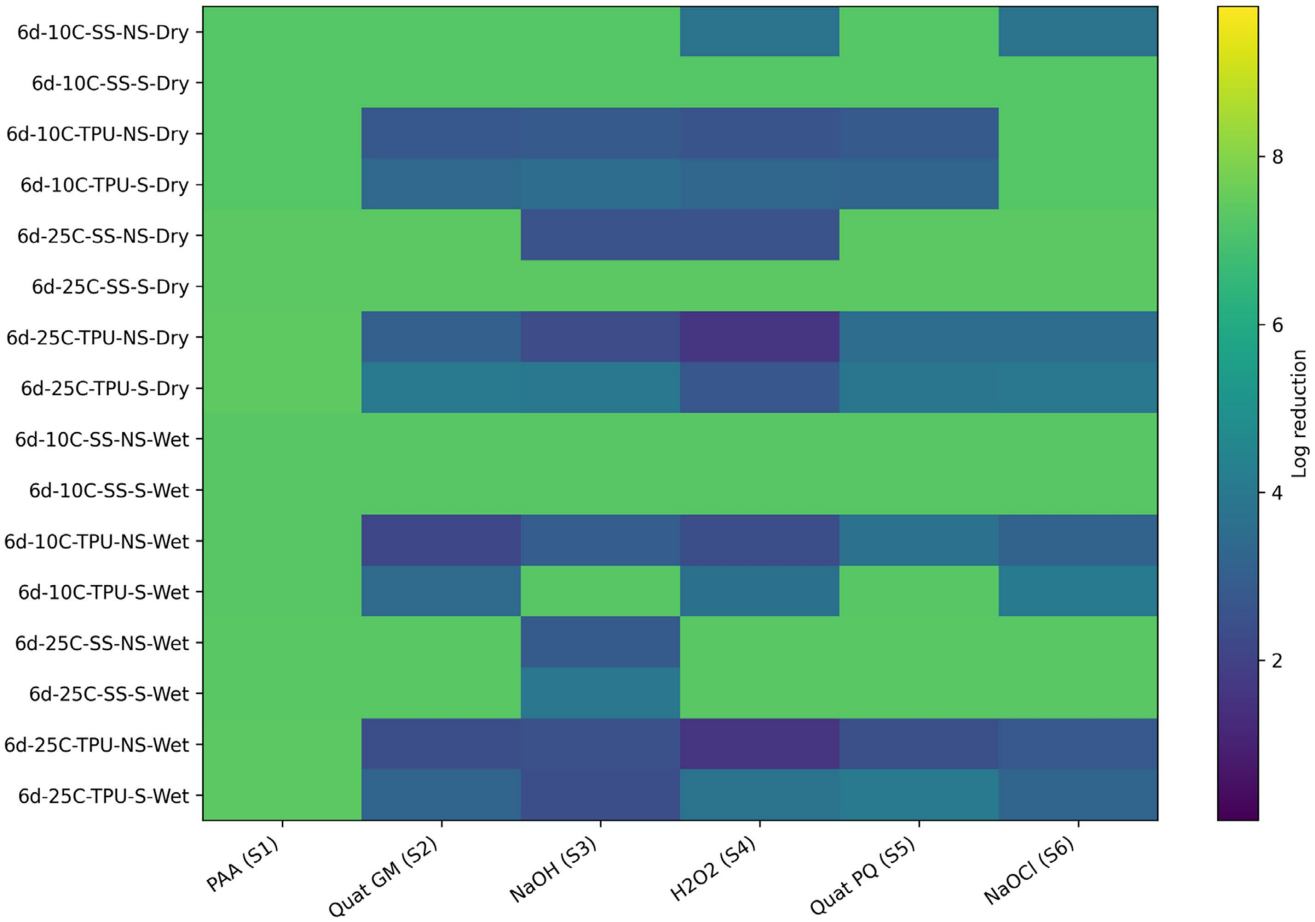
*Heat map* of sanitizer efficacy (log reduction) against multispecies biofilms (T2) - *E. coli* O157:H7. Sanitizers: S1 = peroxyacetic acid (PAA, BioDestroy^®^, 600 ppm); S2 = quaternary ammonium compound (GM, 600 ppm); S3 = sodium hydroxide (2,500 ppm); S4 = hydrogen peroxide (250 ppm); S5 = quaternary ammonium compound (PQ 550 ppm); S6 = sodium hypochlorite (1,200 ppm). Conditions: 6 d storage; 10 °C or 25 °C; stainless steel (SS) or polyurethane (TPU); scrubbed (S) or non-scrubbed (NS); dry vs. wet biofilm surface (*p ≤* 0.05). The color gradient indicates the magnitude of log reduction.

Regarding *E. coli* O157 reductions within the T2 biofilm combination, hydrogen peroxide was the least effective sanitizer across conditions, as evidenced by the predominance of lower log reduction values in the heat map (Figure 11; Table 8). This limited effectiveness aligns with its rapid neutralization within the EPS matrix, reducing its ability to reach embedded bacterial cells (da Cruz Nizer et al., 2024). Sodium hydroxide, Quats (GM and PQ), and sodium hypochlorite exhibited intermediate effectiveness, with performance varying depending on surface type and biofilm condition. In contrast, BioDestroy consistently achieved the highest reductions against O157:H7 across the tested conditions, indicating superior performance.

**Table 8:** Effect of the different sanitizers^1^ applied to multiple pathogen species biofilm formed under different environmental and management conditions, and reduction values are noted for *E. coli* (T2) (*P <* 0.0001; SEM = 0.09).

Condition	Biofilm surface dryness	S1	S2	S3	S4	S5	S6
6d-10C-SS-NS	Dry	7.25 ^A/a/x^	7.25 ^A/a/x^	7.25 ^A/a/x^	3.76 ^B/b/x^	7.25 ^A/a/x^	3.79 ^B/d/x^
6d-10C-SS-S		7.25 ^A/a/x^	7.25 ^A/a/x^	7.25 ^A/a/x^	7.25 ^A/a/x^	7.25 ^A/a/x^	7.25 ^A/a/x^
6d-10C-TPU-NS		7.25 ^A/a/x^	2.77 ^BC/e/x^	2.85 ^B/d/x^	2.61 C^/d/x^	2.82 ^B/e/x^	7.24 ^A/a/x^
6d-10C-TPU-S		7.25 ^A/a/x^	3.42 ^B/c/x^	3.54 ^B/c/x^	3.29 C^/c/x^	3.23 C^/d/x^	7.25 ^A/a/x^
6d-25C-SS-NS		7.33 ^A/a/x^	7.33 ^A/a/x^	2.57 ^B/e/x^	2.52 ^B/d/x^	7.33 ^A/a/x^	7.33 ^A/a/x^
6d-25C-SS-S		7.33 ^A/a/x^	7.33 ^A/a/x^	7.33 ^A/a/x^	7.33 ^A/a/x^	7.33 ^A/a/x^	7.33 ^A/a/x^
6d-25C-TPU-NS		7.37 ^A/a/x^	3.10 ^C/d/x^	2.36 ^D/e/x^	1.64 ^E/e/x^	3.56 ^B/c/x^	3.56 ^B/c/x^
6d-25C-TPU-S		7.37 ^A/a/x^	4.00 ^B/b/x^	3.98 ^B/b/x^	2.72 C^/d/x^	3.88 ^B/b/x^	3.98 ^B/b/x^
6d-10C-SS-NS	Wet	7.26 ^A/a/x^	7.26 ^A/a/x^	7.26 ^A/a/x^	7.26 ^A/a/y^	7.26 ^A/a/x^	7.26 ^A/a/y^
6d-10C-SS-S		7.26 ^A/a/x^	7.26 ^A/a/x^	7.26 ^A/a/x^	7.26 ^A/a/x^	7.26 ^A/a/x^	7.26 ^A/a/x^
6d-10C-TPU-NS		7.27 ^A/a/x^	2.16 ^E/e/y^	3.00 C^/c/x^	2.42 ^D/c/x^	3.72 ^B/c/y^	3.16 C^/c/y^
6d-10C-TPU-S		7.27 ^A/a/x^	3.45 ^D/b/x^	7.27 ^A/a/y^	3.67 C^/b/y^	7.27 ^A/a/y^	4.10 ^B/b/y^
6d-25C-SS-NS		7.31 ^A/a/x^	7.31 ^A/a/x^	2.88 ^B/c/y^	7.31 ^A/a/x^	7.31 ^A/a/x^	7.31 ^A/a/x^
6d-25C-SS-S		7.31 ^A/a/x^	7.31 ^A/a/x^	3.95 ^B/b/y^	7.31 ^A/a/x^	7.31 ^A/a/x^	7.31 ^A/a/x^
6d-25C-TPU-NS		7.36 ^A/a/x^	2.42 C^/d/y^	2.51 C^/d/x^	1.60 ^D/d/x^	2.46 C^/d/y^	2.81 ^B/d/y^
6d-25C-TPU-S		7.36 ^A/a/x^	3.20 ^E/c/y^	2.43 ^D/d/y^	3.75 C^/b/y^	4.02 ^B/b/x^	3.21 ^E/c/y^

^1^Sanitizers: S1 = PAA (Organic peroxy acids or BioDestroy^®^) 600 ppm; S2 = Quats (GM) 600 ppm; S3 = Sodium hydroxide (SHyd) 2,500 ppm; S4 = Hydrogen peroxide (HyP) 250 ppm; S5 = Quats (PQ) = 550 ppm; S6 = Sodium hypochlorite (Shypo) 1,200 ppm. Storing day: 6 days = 6d. Temperature: 10 °C vs. 25 °C. Coupon material: stainless steel = SS, polyurethane = TPU. Biofilm mechanical removal: scrubbed = S, no scrubbed = NS. Controls: Biofilms non-treated with the biocides. A-F/ = indicates significant differences (*P ≤* 0.05) between sanitizers within each environment and management condition combination and biofilm surface dryness. /a-g/= indicates differences (*p ≤* 0.05) between combined conditions for the same biofilm surface dryness and sanitizer. /xy = indicates differences (*p ≤* 0.05) between biofilm surface dryness for the same combined condition and sanitizer.

**Figure 12:**
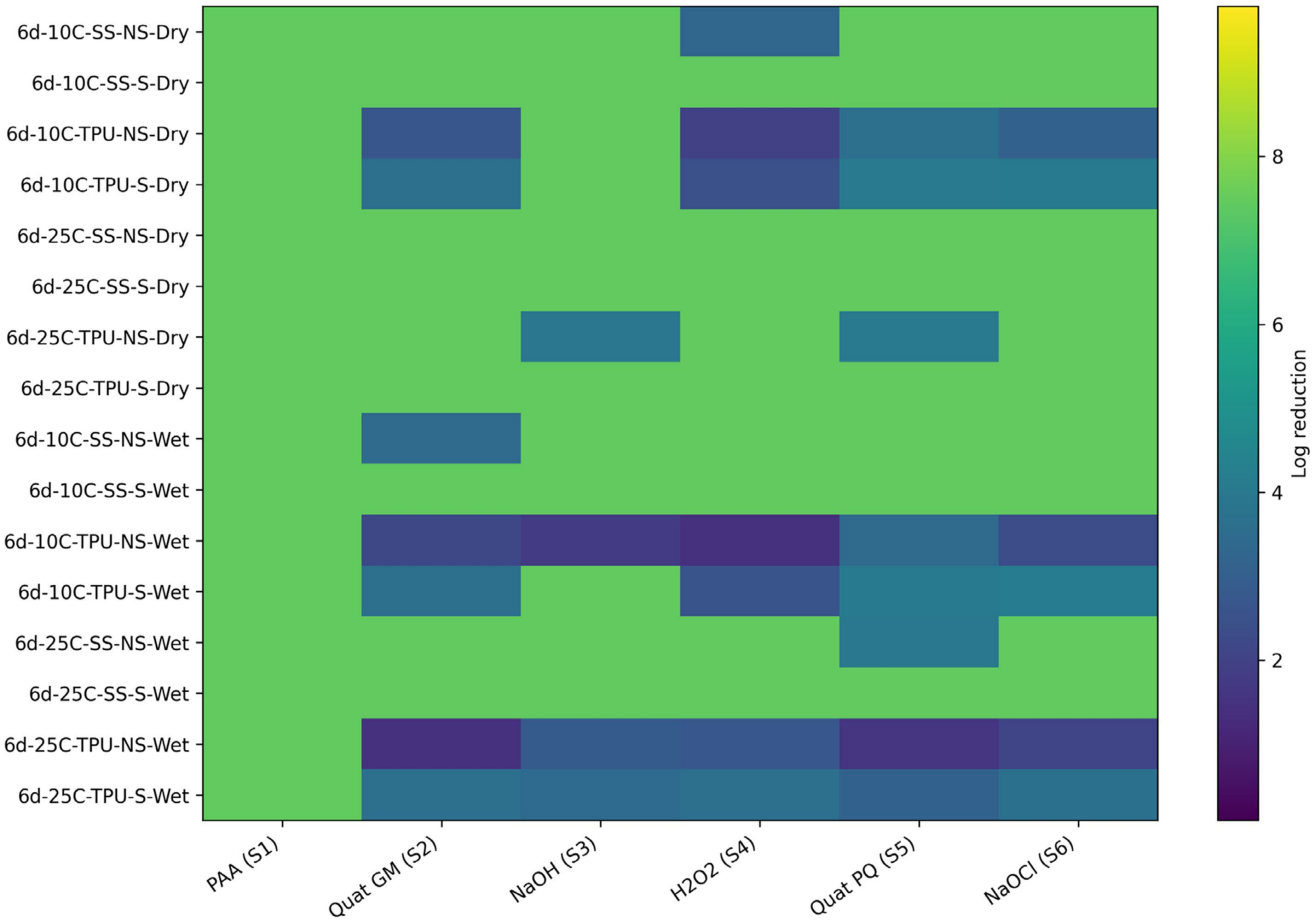
*Heat map* of sanitizer efficacy (log reduction) against multispecies biofilms (T3) - spoilage bacteria. Sanitizers: S1 = peroxyacetic acid (PAA, BioDestroy^®^, 600 ppm); S2 = quaternary ammonium compound (GM, 600 ppm); S3 = sodium hydroxide (2,500 ppm); S4 = hydrogen peroxide (250 ppm); S5 = quaternary ammonium compound (PQ, 550 ppm); S6 = sodium hypochlorite (1,200 ppm). Conditions: 6 d storage; 10 °C or 25C; stainless steel (SS) or polyurethane (TPU); scrubbed (S) or non-scrubbed (NS); dry vs. wet biofilm surface (*p ≤* 0.05). The color gradient indicates the magnitude of log reduction.

Like the T1 combination, TPU surfaces supported greater O157 persistence within the biofilm combination T2, regardless of mechanical removal. This suggests that biofilms formed by the T2 bacterial combination are not easily disrupted on TPU surfaces. The inability of sanitizers to effectively disrupt biofilms on TPU surfaces suggests synergistic interactions between *Comamonas koreensis* and *Raoultella terrigena* within the T2 combination, potentially leading to increased EPS production or structural reinforcement that shields O157 Cells. Sanitizers demonstrated effectiveness on scrubbed SS surfaces with both dry and wet biofilms, except for sodium hydroxide on wet biofilms. The reduced efficacy of sodium hydroxide under these conditions may be attributed to the dilution of its active components or diminished interaction with biofilm elements in the presence of moisture, highlighting the need for protocol optimization (Boyce, 2016; Evangelista et al., 2020). For biofilm combination T3 (antagonistic), *Pseudomonas aeruginosa + C. koreensis* + O157:H7 R508, interestingly, SP within wet biofilms showed more resilience to sanitizers than the dry counterpart (Figure 12; Table 9). Interestingly, dry biofilms formed at 10 °C on TPU surfaces showed poor responsiveness to Quats (GM and PQ), hydrogen peroxide, and sodium hypochlorite. In the case of wet biofilms from the T2 combination, sanitizers were unable to eliminate *Pseudomonas* and *Comamonas* on TPU surfaces at both 10 °C and 25 °C, and mechanical removal had no significant effect. This highlights the role of EPS in shielding the bacteria from sanitizers, as well as the inherent resistance of *Pseudomonas* to antimicrobial agents due to efflux pumps and biofilm-associated tolerance mechanisms (Korany et al., 2018; Hua et al., 2019b). Sanitizers were more effective in reducing SP within wet and dry biofilms formed on SS that were scrubbed.

**Table 9:** Effect of the different sanitizers^1^ applied to multiple pathogen species biofilm formed under different environmental and management conditions, and reduction values are noted for spoilage (T3) (*P <* 0.0001; SEM = 0.05).

Condition	Biofilm surface dryness	S1	S2	S3	S4	S5	S6
6d-10C-SS-NS	Dry	7.46 ^A/a/x^	7.46 ^A/a/x^	7.46 ^A/a/x^	3.28 ^B/b/x^	7.46 ^A/a/x^	7.46 ^A/a/x^
6d-10C-SS-S		7.46 ^A/a/x^	7.46 ^A/a/x^	7.46 ^A/a/x^	7.46 ^A/a/x^	7.46 ^A/a/x^	7.46 ^A/a/x^
6d-10C-TPU-NS		7.46 ^A/a/x^	2.69 ^D/c/x^	7.46 ^A/a/x^	1.95 ^E/d/x^	3.64 ^B/c/x^	3.14 C^/c/x^
6d-10C-TPU-S		7.46 ^A/a/x^	3.64 C^/b/x^	7.46 ^A/a/x^	2.48 ^D/c/x^	4.02 ^B/b/x^	4.10 ^B/b/x^
6d-25C-SS-NS		7.46 ^A/a/x^	7.46 ^A/a/x^	7.46 ^A/a/x^	7.46 ^A/a/x^	7.46 ^A/a/x^	7.46 ^A/a/x^
6d-25C-SS-S		7.46 ^A/a/x^	7.46 ^A/a/x^	7.46 ^A/a/x^	7.46 ^A/a/x^	7.46 ^A/a/x^	7.46 ^A/a/x^
6d-25C-TPU-NS		7.46 ^A/a/x^	7.46 ^A/a/x^	3.94 ^B/b/x^	7.46 ^A/a/x^	4.01 ^B/b/x^	7.46 ^A/a/x^
6d-25C-TPU-S		7.46 ^A/a/x^	7.46 ^A/a/x^	7.46 ^A/a/x^	7.46 ^A/a/x^	7.46 ^A/a/x^	7.46 ^A/a/x^
6d-10C-SS-NS	Wet	7.46 ^A/a/x^	3.44 ^B/c/y^	7.46 ^A/a/x^	7.46 ^A/a/x^	7.46 ^A/a/x^	7.46 ^A/a/x^
6d-10C-SS-S		7.46 ^A/a/x^	7.46 ^A/a/x^	7.46 ^A/a/x^	7.46 ^A/a/x^	7.46 ^A/a/x^	7.46 ^A/a/x^
6d-10C-TPU-NS		7.46 ^A/a/x^	2.18 ^D/d/y^	1.76 ^E/d/y^	1.48\u00B0^F/e/y^	3.46 ^B/c/y^	2.37 C^/d/y^
6d-10C-TPU-S		7.46 ^A/a/x^	3.63 ^D/b/x^	7.46 ^A/a/x^	2.61 ^E/d/y^	4.05 C^/b/x^	4.15 ^B/b/x^
6d-25C-SS-NS		7.46 ^A/a/x^	7.46 ^A/a/x^	7.46 ^A/a/x^	7.46 ^A/a/x^	3.96 ^B/b/y^	7.46 ^A/a/x^
6d-25C-SS-S		7.46 ^A/a/x^	7.46 ^A/a/x^	7.46 ^A/a/x^	7.46 ^A/a/x^	7.46 ^A/a/x^	7.46 ^A/a/x^
6d-25C-TPU-NS		7.46 ^A/a/x^	1.47 ^E/e/y^	2.88 ^B/c/y^	2.71 C^/c/y^	1.58 ^E/e/y^	2.09 ^D/e/y^
6d-25C-TPU-S		7.46 ^A/a/x^	3.62 ^B/b/y^	3.47 C^/b/y^	3.63 ^B/b/y^	3.15 ^D/d/y^	3.66 ^B/c/y^

^1^Sanitizers: S1 = PAA (Organic peroxy acids or BioDestroy^®^) 600 ppm; S2 = Quats (GM) 600 ppm; S3 = Sodium hydroxide (SHyd) 2,500 ppm; S4 = Hydrogen peroxide (HyP) 250 ppm; S5 = Quats (PQ) = 550 ppm; S6 = Sodium hypochlorite (Shypo) 1,200 ppm. Storing day: 6 days = 6d. Temperature: 10°C vs. 25 °C. Coupon material: stainless steel = SS, polyurethane = TPU. Biofilm mechanical removal: scrubbed = S, no scrubbed = NS. Controls: Biofilms non-treated with the biocides. A-F/ = indicates significant differences (*P ≤* 0.05) between sanitizers within each environment and management condition combination and biofilm surface dryness. /a-g/= indicates differences (*p ≤* 0.05) between combined conditions for the same biofilm surface dryness and sanitizer. /xy = indicates differences (*p ≤* 0.05) between biofilm surface dryness for the same combined condition and sanitizer.

**Figure 13:**
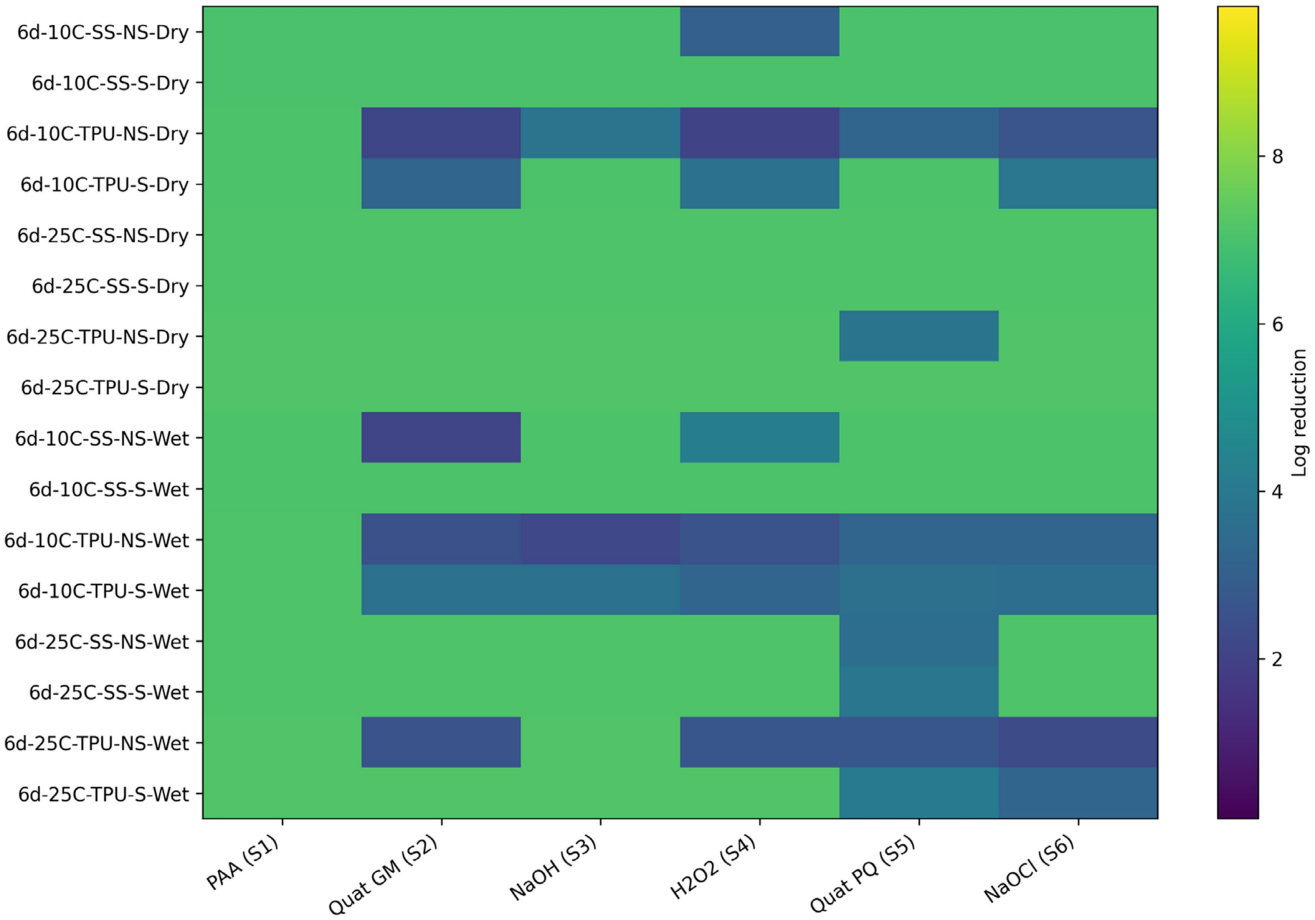
*Heat map* of sanitizer efficacy (log reduction) against multispecies biofilms (T3) - *Escherichia coli*. Sanitizers: S1 = peroxyacetic acid (PAA, BioDestroy^®^, 600 ppm); S2 = quaternary ammonium compound (GM, 600 ppm); S3 = sodium hydroxide (2,500 ppm); S4 = hydrogen peroxide (250 ppm); S5 = quaternary ammonium compound (PQ, 550 ppm); S6 = sodium hypochlorite (1,200 ppm). Conditions: 6 d storage; 10 °C or 25 °C; stainless steel (SS) or polyurethane (TPU); scrubbed (S) or non-scrubbed (NS); dry vs. wet biofilm surface (*p ≤* 0.05). The color gradient indicates the magnitude of log reduction.

Regarding *E. coli* O157 reductions within the T3 combination, it was found that overall, sanitizers were more effective at reducing O157 within dry biofilms that were scrubbed. Dry biofilms, while generally more resistant due to desiccation-induced stress responses, may lose structural integrity when scrubbed, facilitating greater sanitizer penetration (Bridier et al., 2011; da Cruz Nizer et al., 2024). The combination of variables that was more favourable for *E. coli* O157 was on biofilms formed at 10 °C on TPU surfaces that were not scrubbed (Figure 13; Table 10). The persistence of O157 under these conditions could be linked to the cold-induced expression of stress response genes and the protective properties of TPU surfaces (Donlan, 2002; Karavolos et al., 2003).

**Table 10:** Effect of the different sanitizers^1^ applied to multiple pathogen species biofilm formed under different environmental and management conditions, and reduction values are noted for *E. coli* (T3) (*P <* 0.0001; SEM = 0.11).

Condition	Biofilm surface dryness	S1	S2	S3	S4	S5	S6
6d-10C-SS-NS	Dry	7.06 ^A/a/x^	7.06 ^A/a/x^	7.06 ^A/a/x^	3.10 ^B/c/x^	7.06 ^A/a/x^	7.06 ^A/a/x^
6d-10C-SS-S		7.06 ^A/a/x^	7.06 ^A/a/x^	7.06 ^A/a/x^	7.06 ^A/a/x^	7.06 ^A/a/x^	7.06 ^A/a/x^
6d-10C-TPU-NS		7.07 ^A/a/x^	2.13 ^E/c/x^	3.82 ^B/b/x^	2.06 ^E/d/x^	3.23 C^/c/x^	2.66 ^D/c/x^
6d-10C-TPU-S		7.07 ^A/a/x^	3.24 ^D/b/x^	7.07 ^A/a/x^	3.69 C^/b/x^	7.07 ^A/a/x^	3.95 ^B/b/x^
6d-25C-SS-NS		7.13 ^A/a/x^	7.13 ^A/a/x^	7.13 ^A/a/x^	7.13 ^A/a/x^	7.13 ^A/a/x^	7.13 ^A/a/x^
6d-25C-SS-S		7.13 ^A/a/x^	7.13 ^A/a/x^	7.13 ^A/a/x^	7.13 ^A/a/x^	7.13 ^A/a/x^	7.13 ^A/a/x^
6d-25C-TPU-NS		7.16 ^A/a/x^	7.16 ^A/a/x^	7.16 ^A/a/x^	7.16 ^A/a/x^	3.86 ^B/b/x^	7.16 ^A/a/x^
6d-25C-TPU-S		7.16 ^A/a/x^	7.16 ^A/a/x^	7.16 ^A/a/x^	7.16 ^A/a/x^	7.16 ^A/a/x^	7.16 ^A/a/x^
6d-10C-SS-NS	Wet	7.07 ^A/a/x^	2.07 C^/d/y^	7.07 ^A/a/x^	4.21 ^B/b/y^	7.07 ^A/a/x^	7.07 ^A/a/x^
6d-10C-SS-S		7.07 ^A/a/x^	7.07 ^A/a/x^	7.07 ^A/a/x^	7.07 ^A/a/x^	7.07 ^A/a/x^	7.07 ^A/a/x^
6d-10C-TPU-NS		7.10 ^A/a/x^	2.50 C^/c/y^	2.24 ^D/c/y^	2.52 C^/d/y^	3.26 ^B/d/x^	3.26 ^B/c/y^
6d-10C-TPU-S		7.10 ^A/a/x^	3.72 ^B/b/y^	3.72 ^B/b/y^	3.23 C^/c/y^	3.64 ^B/c/y^	3.58 ^B/b/y^
6d-25C-SS-NS		7.11 ^A/a/x^	7.11 ^A/a/x^	7.11 ^A/a/x^	7.11 ^A/a/x^	3.60 ^B/c/y^	7.11 ^A/a/x^
6d-25C-SS-S		7.11 ^A/a/x^	7.11 ^A/a/x^	7.11 ^A/a/x^	7.11 ^A/a/x^	3.89 ^B/b/y^	7.11 ^A/a/x^
6d-25C-TPU-NS		7.15 ^A/a/x^	2.58 ^B/c/y^	7.15 ^A/a/x^	2.69 ^B/d/y^	2.67 ^B/e/y^	2.32 C^/d/y^
6d-25C-TPU-S		7.15 ^A/a/x^	7.15 ^A/a/x^	7.15 ^A/a/x^	7.15 ^A/a/x^	4.10 ^B/b/y^	3.26 C^/c/y^

^1^Sanitizers: S1 = PAA (Organic peroxy acids or BioDestroy^®^) 600 ppm; S2 = Quats (GM) 600 ppm; S3 = Sodium hydroxide (SHyd) 2,500 ppm; S4 = Hydrogen peroxide (HyP) 250 ppm; S5 = Quats (PQ) = 550 ppm; S6 = Sodium hypochlorite (Shypo) 1,200 ppm. Storing day: 6 days = 6d. Temperature: 10°C vs. 25 °C. Coupon material: stainless steel = SS, polyurethane = TPU. Biofilm mechanical removal: scrubbed = S, no scrubbed = NS. Controls: Biofilms non-treated with the biocides. A-F/ = indicates significant differences (*P ≤* 0.05) between sanitizers within each environment and management condition combination and biofilm surface dryness. /a-g/= indicates differences (*p ≤* 0.05) between combined conditions for the same biofilm surface dryness and sanitizer. /xy = indicates differences (*p ≤* 0.05) between biofilm surface dryness for the same combined condition and sanitizer.

However, for Quats (GM), hydrogen peroxide, and sodium hypochlorite, mechanical removal helped reduce O157, but did not completely eliminate it. Biofilms formed on SS responded well to sanitizers, except hydrogen peroxide on non-scrubbed surfaces, showing a reduction of 3.10 out of 7 log10 CFU/cm^2^. For wet T3 biofilms, similar to what was observed for dry biofilms, the combination of TPU surfaces and 10 °C resulted in reduced sanitizer efficacy; mechanical removal did not have a significant impact. This could be attributed to the increased resilience of *Pseudomonas*-dominated biofilms under these conditions, driven by adaptive responses to low temperatures and TPU’s favourable surface characteristics for biofilm formation (Xu et al., 2022; Nan et al., 2023). SS surfaces responded better to sanitizers when the surfaces were scrubbed. Interestingly, the effectiveness of Quats (PQ) was influenced by biofilm moisture levels, being more effective against dry biofilms and less effective against wet biofilms. This moisture-dependent variability in Quats’ performance may be due to altered interaction dynamics between the biofilm components and the sanitizer’s active compounds in different hydration states (Centeleghe et al., 2022).

### 3.3 Relative cleanliness of coupon contact surfaces after biocide exposure

#### 3.3.1 *E. coli* O157 survival within the different biofilm combinations

The survival of *E. coli* O157:H7 in both single-species and multispecies biofilms was more pronounced on TPU and SS surfaces that were not scrubbed, particularly in wet and dry biofilms formed at 25 °C. In wet single-species biofilms, O157:H7 exhibited 88% survival at 25 °C following treatment of 6-d-old biofilms with hydrogen peroxide, while no survival was detected at 30 and 60 d (Table 11). When examining dry single-species biofilms, survival was only observed at 25 °C on non-scrubbed surfaces, primarily for 6-d-old biofilms. Survival rates ranged from 22.2 to 88.8%. The less effective sanitizers were sodium hypochlorite, hydrogen peroxide and Quats, and TPU was the material that allowed more survival (Table 11).

**Table 11:** Consolidated survival percentage of wet and dry STEC O157:H7 R5O8 single-species biofilms in modified TSB.

Humidity condition	Cleaning condition	Days	Temperature (°C)	Coupon	Sanitizers	STEC survival % (n/N)
WET	NS	6	25	TPU	Hydrogen peroxide	88.88 (8/9)
DRY	NS	30	25	SS	Sodium hypochlorite	44.44 (4/9)
	NS	6	25	TPU	Quats (GM)	44.44 (4/9)
	NS	6	25	SS	Quats (GM)	77.77 (7/9)
	NS	6	25	TPU	Hydrogen peroxide	55.55 (5/9)
	NS	6	25	TPU	Quats (PQ)	88.88 (8/9)
	NS	6	25	TPU	Sodium hypochlorite	33.33 (3/9)
	NS	6	25	SS	Sodium hypochlorite	22.22 (2/9)
	NS	30	25	TPU	Sodium hypochlorite	22.22 (2/9)
	NS	60	25	TPU	Sodium hypochlorite	33.33 (3/9)

Survival was performed by enrichment in mTSB after treatment with organic peroxy acids (BioDestroy^®^); Quats (GM), sodium hydroxide, hydrogen peroxide, Quats (PQ), and sodium hypochlorite.

For multispecies biofilms in combination with T1, survival was observed only in dry biofilms formed at 25 °C (Table 12). The highest survival rates were observed on TPU coupons in 30-day-old biofilms, with survival rates ranging from 33.3 to 100%. The highest survival rate of 100% was observed on 30-d biofilms formed on TPU and treated with sodium hypochlorite (Table 12). Biofilm combination T1, consisting of *Carnobacterium piscicola* and *Lactobacillus bulgaricus* + O157:H7, exhibited no O157:H7 survival at 10 °C (data not shown).

**Table 12:** Survival percentage of O157:H7 R5O8, after 24 h enrichment from dry multispecies biofilms (T1: *Carnobacterium piscicola + Lactobacillus Bulgaricus,* T2: *Comamonas koreensis + Raoultella terrigena* and T3: *Pseudomonas aeruginosa + C. koreensis*) after treatment with organic peroxy acids (BioDestroy^®^); Quats (GM and PQ), sodium hydroxide, hydrogen peroxide and sodium hypochlorite and stored at 25 or 10°C for 6, 30 and 60 days.

Biofilm	Cleaning condition	Days	Temperature (°C)	Coupon	Sanitizers	STEC survival % (n/N)
T1	S	6	25	TPU	Quats (PQ)	33.33 (3/9)
	S	30	25	TPU	Quats (PQ)	44.44 (4/9)
	NS	30	25	TPU	Quats (PQ)	66.66 (6/9)
	NS	30	25	TPU	Sodium hypochlorite	100.0 (9/9)
	NS	30	25	SS	Sodium hypochlorite	33.33 (3/9)
T2	S	30	25	TPU	Quats (PQ)	44.44 (4/9)
	NS	30	25	SS	Quats (PQ)	88.88 (8/9)
	NS	30	10	TPU	Quats (PQ)	55.55 (5/9)
	NS	30	10	TPU	Sodium hypochlorite	33.33 (3/9)
	NS	30	10	TPU	Quats (GM)	44.44 (4/9)
	NS	60	25	TPU	Quats (PQ)	22.22 (2/9)
T3	NS	6	25	SS	Quats (GM)	55.55 (5/9)
	NS	6	25	SS	Quats (PQ)	66.66 (6/9)
	NS	6	25	TPU	Sodium hydroxide	33.33 (3/9)
	NS	6	25	TPU	Hydrogen peroxide	44.44 (4/9)
	NS	6	25	TPU	Sodium hypochlorite	77.77 (7/9)
	NS	30	25	TPU	Hydrogen peroxide	44.44 (4/9)
	NS	30	25	SS	Hydrogen peroxide	33.33 (3/9)
	NS	30	10	SS	Sodium hypochlorite	55.55 (5/9)
	NS	60	25	TPU	Hydrogen peroxide	33.33 (3/9)
	NS	60	10	TPU	Quats (GM)	22.22 (2/9)

Biofilm combination T2, consisting of *Comamonas koreensis* and *Raoultella terrigena*, supported O157:H7 survival only at 25 °C on 30-day-old wet biofilms. No survival was detected at either 6 or 60 days at either 10 °C or 25 °C. The highest survival rate of 66% was found on 30-day-old biofilms formed on TPU surfaces treated with Quats (GM) (Table 13). At 10 °C, O157 survival was observed to be 22.2% within the combination T2 after 30 days on wet biofilms on SS when exposed to Quats (GM) (Tables 6, 13). Interestingly, when looking at the T2 dry biofilm combination, O157 was able to survive more often on 30-d biofilms formed on TPU at 25 and 10 °C. Survival reached 88.88% on SS treated with Quats (PQ) under non-scrubbed conditions, compared to 44.44% on TPU that was scrubbed, at 30 days and 10 °C survival on TPU under non-scrubbed conditions varied by sanitizer. At 30 days and 10 °C, survival of O157:H7 on TPU surfaces varied depending on whether the surfaces were scrubbed or not, and also differed between sanitizers. Under non-scrubbed conditions, quaternary ammonium compounds (PQ and GM) showed moderate survival rates (55.55 and 44.44%, respectively), while sodium hypochlorite reduced survival further to 33.33% (Tables 5, 12). Scrubbing generally lowered survival, indicating that both sanitizer type and mechanical removal influenced efficacy.

**Table 13:** Survival percentage of O157:H7 R5O8, after 24 h enrichment from wet multispecies biofilms (T1: *Carnobacterium piscicola + Lactobacillus Bulgaricus,* T2: *Comamonas koreensis + Raoultella terrigena* and T3: *Pseudomonas aeruginosa + C. koreensis*) after treatment with organic peroxy acids (BioDestroy^®^); Quats (GM); Sodium hydroxide; Hydrogen peroxide; Quats (PQ); Sodium hypochlorite and stored at 25 or 10°C for 6, 30 and 60 days.

Biofilm	Cleaning condition	Days	Temperature (°C)	Coupon	Sanitizers	STEC survival % (n/N)
T1	No Survival					
T2	NS	30	25	TPU	Quats (GM)	66.66 (6/9)
	NS	30	25	TPU	Hydrogen peroxide	44.44 (4/9)
	NS	30	10	SS	Quats (GM)	22.22 (2/9)
T3	NS	6	25	SS	Quats (GM)	33.33 (3/9)
	NS	30	25	TPU	Hydrogen peroxide	22.22 (2/9)
	NS	60	10	SS	Quats (GM)	22.22 (2/9)
	NS	60	25	TPU	Hydrogen peroxide	44.44 (4/9)

At 60 days and 25 °C, survival was considerably decreased on non-scrubbed TPU treated with Quats (PQ), dropping to 22.22% (Table 12), suggesting that prolonged storage time and increased temperature had a significant impact on reducing O157:H7 persistence.

In the case of biofilm combination T3, which involved *Pseudomonas aeruginosa* and *Comamonas koreensis*, the highest survival rates were observed in 60-day-old wet biofilms, 44% on TPU surfaces at 25 °C, when treated with hydrogen peroxide. Notable survival rates for T3 + O157:H7 were 33% on non-scrubbed SS surfaces at 6 days at 25 °C and 60 days at 10 °C when treated with quaternary ammonium compounds (Quats) (GM) (Tables 6, 13). Interestingly, 30-day-old T3 + O157:H7 biofilm at 25 °C showed survival of 22% when treated with hydrogen peroxide (Table 13). On the other hand, T3 dry biofilm combination showed survival on 6, 30 and 60 days when the TPU and SS surfaces were not scrubbed at 10 °C and 25 °C. The highest survival was found at 25 °C on day 6 when treated with sodium hypochlorite and Quats (PQ), with 77 and 66% survival rate. Interestingly, 30 days (44%) and 60 days (33%) old T3 dry biofilms survived at 25 °C when treated with hydrogen peroxide. It was surprising to note that 10 °C dry T3 biofilms survived on day 30 (SS) and 60 (TPU) when treated with sodium hypochlorite and Quats (GM) with a survival rate of 55 and 22% (Table 12).

#### 3.3.2 ATP testing on coupons

Data showed significant differences in ATP bioluminescence readings (RLU) between scrubbed and non-scrubbed surfaces (Refer to Supplementary Table S2). Overall, on scrubbed surfaces, the average RLU values for wet biofilms were 171.80, while they were slightly lower (125.7 RLU) for dry biofilms. In contrast, non-scrubbed biofilms exhibited substantially higher RLU readings, averaging 15,635.65 for wet biofilms and 14,463.8 for dry biofilms, reflecting organic contamination. When examining specific surface types, scrubbed SS surfaces (wet 197.1, dry 87.5 RLU) yielded better results for dry biofilms compared to TPU surfaces (wet 151.09 and dry 139.0 RLU). Both materials exhibited significantly higher RLU values on non-scrubbed surfaces. The results showed some differences in ATP bioluminescence readings (RLU) by temperature. At 10 °C, scrubbed surfaces averaged 181.2 RLU for wet biofilms and 147.5 RLU for dry biofilms. In contrast, non-scrubbed surfaces exhibited higher values, with wet biofilms averaging 14,069.4 RLU and dry biofilms averaging 11,625.22 RLU. At 25 °C, scrubbed surfaces recorded an average of 182.21 RLU for wet biofilms and 97.30 RLU for dry biofilms. Non-scrubbed surfaces again showed much higher RLU values, with wet biofilms averaging 17,354.37 RLU and dry biofilms 14,661 RLU (Refer to Supplementary Table S2).

The results of the biofilm combination showed differences, mainly when they were scrubbed. The T1 wet-biofilm combination was the least resilient (wet 30 and dry 20.67 RLU). For T2, readings for wet biofilms were 120.67 for wet and 80.6 RLU for dry biofilms. For combination T3, the residual contamination was higher: 357.4 for wet biofilms and 338 RLU for dry biofilms (Refer to Supplementary Table S2).

These findings reinforce that dry multispecies biofilms, especially when not mechanically disrupted, support greater O157:H7 survival—likely due to their structural and physiological resilience, as described in previous literature (Bridier et al., 2011; O’Reilly et al., 2025).

While surface scrubbing may aid in reducing biofilm mass, the residual survival of *E. coli* O157:H7, particularly after hydrogen peroxide treatment, could also be influenced by the bacterium’s genetic tolerance to oxidative stress (*kat*G, *oxy*R, *sod*A) and the poor penetration of peroxide through EPS matrices (Poole, 2012). These factors may have limited the additional benefit of mechanical removal under peroxide treatment conditions (Poole, 2012).

## 4 Conclusion

Overall, this research demonstrates that biofilm control strategies must be tailored to specific conditions, including surface type, biofilm age, microbial composition, temperature, and moisture level. Mechanical removal should be integrated as a critical step prior to sanitizer application, as visually clean surfaces are not necessarily microbiologically safe. These findings highlight the importance of objective hygiene assessments beyond visual inspection.

The dynamic resistance profiles of biofilms show the need for multi-tiered sanitation protocols (mechanical removal, chemical action, environment control), particularly targeting early-stage biofilms (6 days), low-temperature conditions (10 °C), and SS surfaces. Mechanical scrubbing can further disrupt biofilm integrity and enhance the activity of biocides. Among the sanitizers tested, sodium hypochlorite and BioDestroy^®^ were the most effective. In addition, co-culturing with lactic acid bacteria (*Carnobacterium piscicola* + *Lactobacillus bulgaricus*) enhanced *E. coli* O157 reduction and limited survival.

*E. coli* O157:H7 survival was highest under dry conditions, at 25 °C, on TPU surfaces that were not scrubbed, particularly when sanitizers with reduced efficacy (quats, sodium hypochlorite, hydrogen peroxide) were used.

Spoilage (SP) and LAB organisms showed distinct resilience patterns. LAB, *Carnobacterium piscicola* and *Lactobacillus bulgaricus* were the easiest to eliminate, with sodium hypochlorite showing the only limitation on TPU. *Comamonas koreensis* and *Raoultella terrigena* were harder to remove, particularly in dry biofilms and on TPU, though BioDestroy^®^ was effective across conditions. *Pseudomonas aeruginosa* was the most resilient, especially in wet TPU biofilms at low temperature, highlighting the difficulty of eliminating multispecies biofilms when this organism is present. These organisms not only drive quality losses (e.g., reduced shelf life) but can also shelter pathogens within mixed biofilms, elevating food-safety risk.

These findings emphasize the importance of explicitly incorporating biofilm management into cleaning and sanitation procedures. By addressing biofilm-related risks directly, sanitation standard operating procedures (SSOPs) can more effectively mitigate contamination and improve overall food safety.

## Data Availability

The raw data supporting the conclusions of this article will be made available by the authors, without undue reservation.
