# Supplementary material for: Efficacy of chemical sanitizers against E. coli O157:H7 in single- and multi-species biofilms under wet and dry conditions
Source: Front Microbiol. 2026 Feb 27;17:1682881. doi: 10.3389/fmicb.2026.1682881 (PMC12982456; doi:10.3389/fmicb.2026.1682881)
Supplement: Supplementary file 1 [file Table_1.docx]

| **Table S1.** *Biocides and manufacture recommended concentrations.* | | | | | | | |
| --- | --- | --- | --- | --- | --- | --- | --- |
| **Trade Name** | **Active Ingredients** | **Acronyms** | **Stock** | **Dilution** | **Use Level** | **Contact time** | **Temperature** |
| Chloreco | Sodium hypochlorite | Shypo | 12% | 1:100 | Sanitizing: 200 ppm  (no rinse needed) | < 5 vary min | 5 - 65°C |
|  |  |  |  |  | Surface sanitizing: 12000 to 120000 ppm (must be rinsed with water) | 5 – 30 min |  |
| Caustek 50 | Sodium hydroxide | Shyd | 50% | 1:200 – 1:9 | 2500 ppm (must be rinsed with water) | 15-40 min. | 50 - 100°C  (Optimal: 75-85°C) |
| Powerquat | Quaternary ammonium,C12-18-alkyl[(ethyl phenyl)methyl] dimethyl, chlorides (5%) | Quats (PQ) | 10% | 22:4000 | 200 ppm (no rinse)  550 ppm | 10 min | Not specified |
|  | Quaternary ammonium, benzyl-C12-18-alkyl dimethyl, chlorides and ethanol (5%) |  |  |  |  |  |  |
| Oxygerm | Hydrogen peroxide 21.7% Peracetic acid 5.1.% | HyP | 5% | 1:200 | 250 ppm (must be rinsed) | Vary | 5 - 40°C |
| Germarc | Quaternary ammonium compounds, benzyl-C12-16-alkyl dimethyl, chlorides and ethanol | Quats (GM) | 10% | 1:160 | Sanitation: 200 ppm  Disinfection: 400-600 ppm | Up to 10 min | 5 - 65°C |
| BioDestroy® | Hydrogen peroxide, dodecylbenzene sulphonic acid, Acetic acid, Peroxyacetic acid Alcohols, C12-15, ethoxylated | PAA | 6% | 1:100 | 600 ppm | 5 min | Not specified |
| *All sanitizers were obtained from SANI MARC^®^. Two types of Quats are used for this experiment, PowerQuat and Germarc. PowerQuat is specially designed for food processing plants where disinfection is of prime importance and mostly used on hard surfaces, and proved to be an effective bactericide in the presence of organic soil. In contrast, Germarc is commonly used as a sanitizer on all surfaces with N-Alkyl (50% C14, 40% C12, 10% C16) dimethyl benzyl ammonium chloride: 10.0% and its effectiveness is affected by the presence of organic soils and needs higher exposure time. | | | | | | | |

| **Table S2** ATP reading in RLU on TPU and SS coupons before and after scrubbing | | | | | | |
| --- | --- | --- | --- | --- | --- | --- |
| **Biofilm** | **Humidity condition** | **Coupons** | **Temperature(°C)** | **Day** | **Not-scrubbed** | **Scrubbed** |
| T1 | WET | TPU | 25 | 6 | 24340 | 18 |
|  |  | TPU | 10 | 6 | 14700 | 34 |
|  |  | SS | 25 | 6 | 15320 | 29 |
|  |  | SS | 10 | 6 | 9479 | 89 |
|  |  | TPU | 25 | 30 | 19579 | 18 |
|  |  | SS | 25 | 30 | 16575 | 17 |
|  |  | TPU | 25 | 60 | 10476 | 5 |
|  | DRY | TPU | 25 | 6 | 22390 | 43 |
|  |  | TPU | 10 | 6 | 8720 | 10 |
|  |  | SS | 25 | 6 | 18438 | 42 |
|  |  | TPU | 25 | 30 | 12564 | 13 |
|  |  | SS | 25 | 30 | 9580 | 13 |
|  |  | TPU | 25 | 60 | 9043 | 3 |
| T2 | WET | TPU | 25 | 6 | 24432 | 87 |
|  |  | SS | 25 | 6 | 31653 | 92 |
|  |  | SS | 10 | 6 | 17694 | 119 |
|  |  | TPU | 25 | 30 | 14286 | 196 |
|  |  | SS | 25 | 30 | 6575 | 17 |
|  |  | SS | 25 | 30 | 8580 | 213 |
|  | DRY | TPU | 25 | 6 | 19759 | 110 |
|  |  | TPU | 10 | 6 | 28720 | 54 |
|  |  | SS | 25 | 6 | 18638 | 109 |
|  |  | TPU | 25 | 30 | 11864 | 113 |
|  |  | TPU | 25 | 60 | 7043 | 17 |
| T3 | WET | TPU | 25 | 6 | 16327 | 487 |
|  |  | TPU | 10 | 6 | 12680 | 137 |
|  |  | SS | 10 | 6 | 8658 | 332 |
|  |  | SS | 25 | 6 | 18654 | 333 |
|  |  | TPU | 10 | 30 | 6541 | 174 |
|  |  | TPU | 25 | 30 | 27436 | 593 |
|  |  | SS | 25 | 60 | 8728 | 446 |
|  | DRY | TPU | 25 | 6 | 17291 | 510 |
|  |  | TPU | 10 | 6 | 15720 | 554 |
|  |  | SS | 10 | 6 | 7322 | 186 |
|  |  | TPU | 10 | 30 | 9865 | 102 |
| Note: T1:***Carnobacterium piscicola + Lactobacillus Bulgaricus, T2***: ***Comamonas koreensis + Raoultella terrigena*** and T3: ***Pseudomonas aeruginosa + C. koreensis*** with R5O8 cultured as biofilms, and ATP readings before and after cleaning were measured in RLU. The analysis was done in triplicates, and the mean values before and after scrubbing were reported. | | | | | | |
